# Hospital and municipal wastewater as a source of carbapenem-resistant *Acinetobacter baumannii* and *Pseudomonas aeruginosa* in the environment: a review

**DOI:** 10.1007/s11356-024-34436-x

**Published:** 2024-07-25

**Authors:** Magdalena Męcik, Kornelia Stefaniak, Monika Harnisz, Ewa Korzeniewska

**Affiliations:** Department of Water Protection Engineering and Environmental Microbiology, Faculty of Geoengineering, University of Warmia and Mazury in Olsztyn, Prawocheńskiego 1, 10-720 Olsztyn, Poland

**Keywords:** Hospital wastewater, Environment, Carbapenems, Antibiotic resistance, ARGs, *Acinetobacter baumannii*, *Pseudomonas aeruginosa*

## Abstract

The increase in the prevalence of carbapenem-resistant Gram-negative bacteria, in particular *Acinetobacter baumannii* (CRAB) and *Pseudomonas aeruginosa* (CRPA), poses a serious threat for public health worldwide. This article reviews the alarming data on the prevalence of infections caused by CRAB and CRPA pathogens and their presence in hospital and municipal wastewater, and it highlights the environmental impact of antibiotic resistance. The article describes the key role of antibiotic resistance genes (ARGs) in the acquisition of carbapenem resistance and sheds light on bacterial resistance mechanisms. The main emphasis was placed on the transfer of ARGs not only in the clinical setting, but also in the environment, including water, soil, and food. The aim of this review was to expand our understanding of the global health risks associated with CRAB and CRPA in hospital and municipal wastewater and to analyze the spread of these micropollutants in the environment. A review of the literature published in the last decade will direct research on carbapenem-resistant pathogens, support the implementation of effective preventive measures and interventions, and contribute to the development of improved strategies for managing this problem.

**Graphical Abstract:**

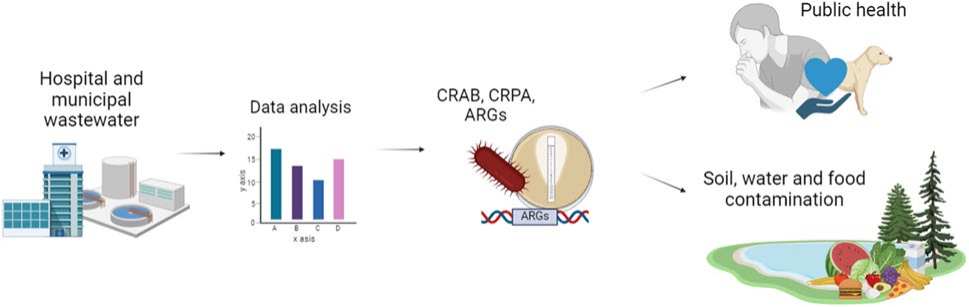

**Supplementary Information:**

The online version contains supplementary material available at 10.1007/s11356-024-34436-x.

## Introduction

Antibiotic resistance is a global problem that emerged ever since antibiotics have been introduced to human and veterinary medicine, food production, and other industries that rely on microorganisms in technological processes. Recent research has shown that pathogens are increasingly resistant to carbapenems, a group of antibiotics that are structurally similar to β-lactams, which gives particular cause for concern (El-Gamal et al. 2017). Carbapenems are considered last-resort antibiotics because they are effective in the treatment of complicated infections. Antibiotic resistance genes (ARGs) that are exchanged by microorganisms via mobile genetic elements (MGEs) and horizontal gene transfer (HGT) play a key role in the spread of antibiotic resistance. Carbapenem-resistant *Acinetobacter baumannii* and *Pseudomonas aeruginosa* bacteria have been classified as priority pathogens that will pose a challenge for the pharmaceutical industry and a serious threat for public health in the future (World Health Organization (WHO) (2017b)). Hospital and municipal wastewater is abundant in antimicrobials, and it will contribute to the spread of carbapenem resistance and exacerbate the associated risks for both humans and the environment (Aubertheau et al. 2017; Rozman et al. 2020). The aim of this study was to analyze the prevalence of carbapenem-resistant *Acinetobacter baumannii* and *Pseudomonas aeruginosa* in hospital wastewater (HWW), municipal wastewater, and the environment based on the latest research findings. A review of the literature published in the last decade will direct research on carbapenem-resistant pathogens, support the implementation of effective preventive measures, and contribute to the development of improved strategies for monitoring this problem.

### Antibiotic resistance worldwide and the role of MGEs in the transmission of ARGs

Growing levels of antibiotic resistance pose a serious threat for public health and the environment. Different definitions of antibiotic resistance have been proposed, depending on the origin and type of microorganisms. All antibiotic-resistant bacteria (ARB) harbor cellular mechanisms that are responsible for tolerance to antimicrobials (Davison et al. 2000). Microbial competition for substrates is the key factor that has been driving the evolution of bacterial resistance mechanisms since ancient times (Frieri et al. 2017). The production of natural secondary metabolites that resemble modern antibiotics is an example of evolutionary adaptation. The introduction of antibiotics into clinical use led to profound changes in microbial evolution and the spread of drug resistance. Antimicrobials exert selective pressure, in particular on microorganisms that are a part of human and animal microbiota, as well as on microorganisms that colonize environments heavily polluted with antibiotics (Larsson and Flach 2022). Antibiotic resistance poses a global challenge due to growing levels of morbidity and mortality in both human and animal populations. The emergence of multidrug-resistant (MDR) Gram-positive and Gram-negative microorganisms has undermined the efficacy of antimicrobial drugs, and some infections are becoming difficult or even impossible to treat (Frieri et al. 2017). At present, antibiotics are used primarily in human and veterinary medicine. Antibiotic consumption was relatively low at the turn of the twentieth and twenty-first centuries (between 1997 and 2007), but it increased considerably in the following years, excluding in countries with limited access to antimicrobials (Bruyndonckx et al. 2021). Growing antibiotic consumption is directly correlated with the increase in mortality due to MDR pathogens (World Health Organization (WHO), 2018). Each year, infections caused by antibiotic-resistant strains are responsible for more than 5 million global deaths (Inda-Díaz et al. 2023). By 2050, the mortality burden of MDR pathogens is projected to increase by 10 million annually, and it will exceed the total number of cancer deaths (Theuretzbacher et al. 2020). Global antibiotic consumption increased by 65% between 2000 and 2015 (Babiecki 2022). At the same time, a minor decrease in antibiotic consumption was noted in high-income countries between 2013 and 2017. However, global antibiotic use is presently expected to increase by 2030 (Batista et al. 2020). According to Kumar et al. (2020), the global use of antimicrobials in the production of animal-based foods (milk, eggs, and meat) was estimated at 63,151 ± 1560 megagrams (Mg) in 2010, with a projected 67% increase to 105,596 ± 3605 Mg by 2030. This increase will be largely determined by the implementation of intensive farming practices and systems. In Asia, antibiotic consumption in livestock farming could reach 51,851 Mg by 2030, marking an increase of 82% from 2010. The overall use of antibiotics in Brazil, Russia, India, China, and South Africa (BRICS) increased by 76% between 2000 and 2010 (Van Boeckel et al. 2017). By 2030, antibiotic consumption in BRICS countries can increase by as much as 99% relative to 2010 (Kumar et al. 2020). In addition, more than 60% of antimicrobials consumed worldwide in recent years had been administered without a prescription, which clearly indicates that rational management of antibiotics poses a considerable problem (Batista et al. 2020). The development and introduction of new therapeutics is a long process, and antibiotic resistance increases at a faster rate than the availability of novel drugs. Bacteria rapidly acquire adaptive traits, including resistance to antibiotics. Extremely drug-resistant (XDR) and totally drug-resistant (TDR) phenotypes (mostly Gram-negative bacteria) that are insensitive to nearly all antimicrobials pose the greatest threat. High-risk MDR clones can lead to rapid diffusion of epidemics due to cross infection and global spread (Mobarki et al. 2019). The One Health approach advocates rational use and dosage of antibiotics because high consumption of antimicrobials significantly contributes to microbial resistance and, consequently, affects the environment (Jiménez-Belenguer et al. 2023).

Bacteria are characterized by both innate resistance, which has evolved in the process of adapting to a given environment, as well as acquired resistance which is associated with the emergence of new defense mechanisms or the exchange of resistance genes between microorganisms via HGT and MGEs (Babiecki 2022). Antibiotic resistance genes (ARGs) are the key molecular mechanism of drug resistance in bacteria. More than 20,000 ARGs have been identified to date (Inda-Díaz et al. 2023). Research on ARGs focuses mainly on environments where antibiotics are frequently used, including hospitals, veterinary clinics, food production, and livestock farms (Tiedje et al. 2019). The relative abundance of antibiotic-resistant strains continues to increase due to the diversity of antimicrobial drugs, their mechanisms of action, and frequent consumption. Bacterial ARGs can encode resistance to various classes of antibiotics, and they can be divided into groups that confer resistance to tetracyclines (*tet*), sulfonamides (*sul*), β-lactams (*bla*), macrolides (*erm*), aminoglycosides (*aac*), fluoroquinolones (*fca*), colistin (*mcr*), vancomycin (*van*), and multiple drugs (MDR) (Jian et al. 2021). The spread of ARGs is directly influenced by their location on MGEs such as plasmids, integrons, and transposons. Research studies analyzing animal manure, sewage sludge, and microbiota colonizing the human digestive tract have demonstrated that ARGs localized on MGEs can be transmitted to other environments via HGT (Kim et al. 2021). Transformation, transduction, and conjugation mechanisms facilitate the exchange of ARGs across ARB and drug-sensitive strains in their respective environments (Partridge et al. 2018; Larsson and Flach 2022).

### Definition of carbapenem antibiotics

**Fig. 1:**
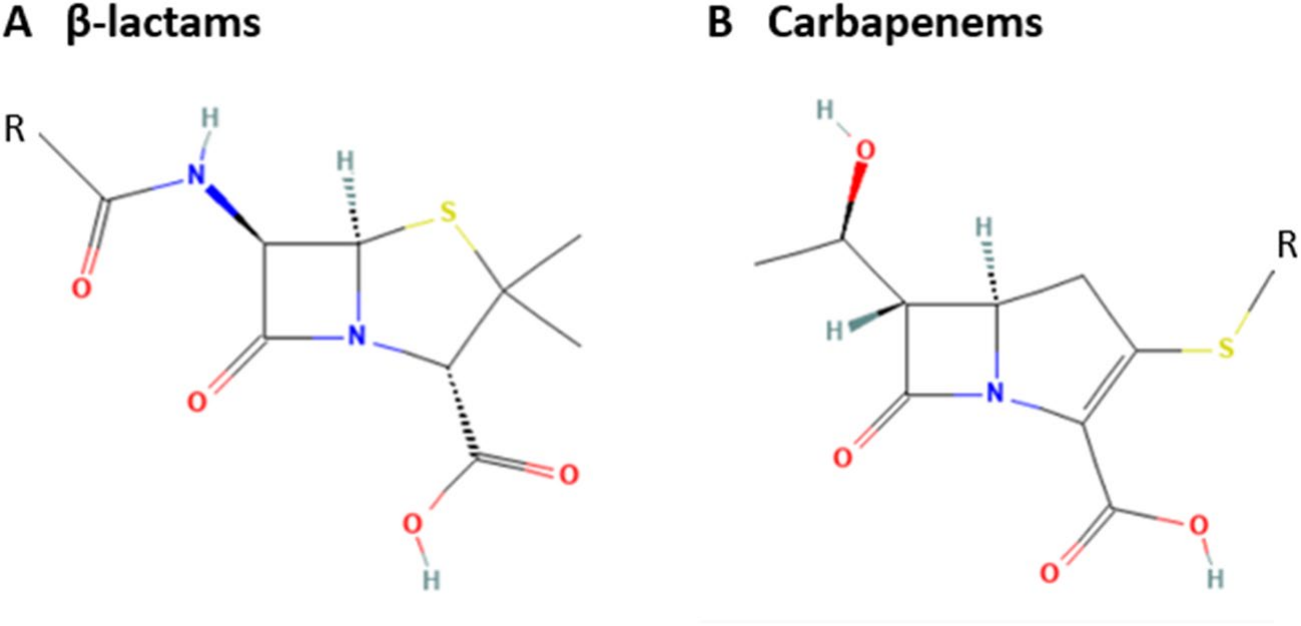
Structural composition of β-lactam and monobactam antibiotics. Core structure of penicillins (A) and core structure of carbapenems based on imipenem (B). R represents side chains that differ among members of the same class of β-lactam and carbapenem antibiotics. Source: PubChem Database with own modifications (https://pubchem.ncbi.nlm.nih.gov/). Created with MS Office

Antibiotics can be divided into several classes based on their mechanism of action and origin. Carbapenems are a large group of antimicrobials that have been introduced into clinical use in the 1980s (Birnbaum et al. 1985). Carbapenems are renowned for their efficacy, and they belong to the class of β-lactam antibiotics which are structurally similar to penicillins (Fig. 1A) and cephalosporins. Carbapenems (Fig. 1B) are derivatives of thienamycin (imipenem), an antibiotic produced by *Streptomyces cattleya* bacteria that naturally colonize soil (El-Gamal et al. 2017).

**Fig. 2:**
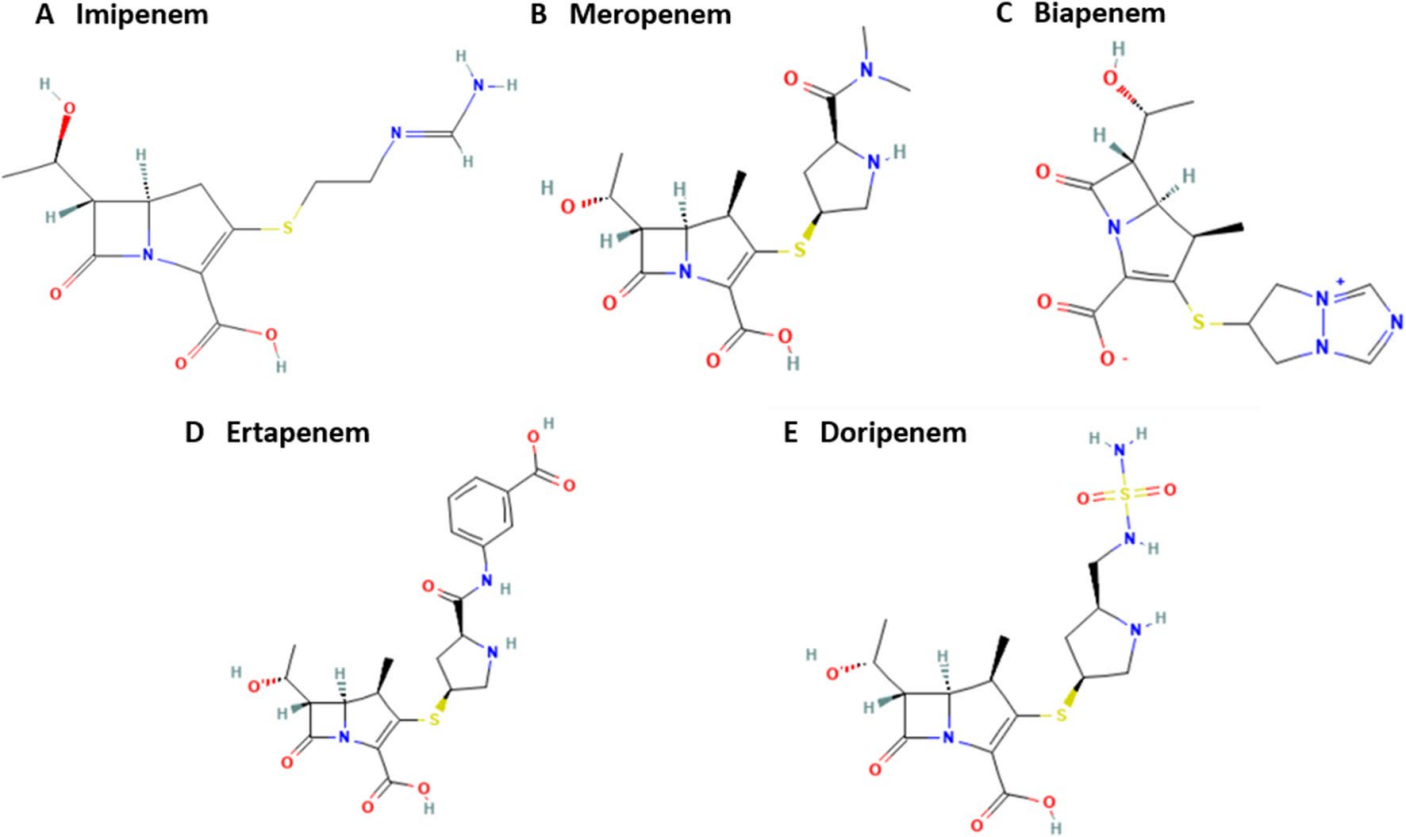
Chemical structure of older carbapenem antibiotics: (A) imipenem; (B) meropenem; (C) biapenem; (D) ertapenem; and (E) doripenem. Source: PubChem Database with own modifications (https://pubchem.ncbi.nlm.nih.gov/). Created with MS Office

Carbapenems are β-lactam antibiotics with the broadest spectrum of activity against pathogens, mainly Gram-negative aerobic bacteria (Hawkey and Livermore 2012; Jeon et al. 2015). Older-generation carbapenems include imipenem, meropenem, biapenem, ertapenem, and doripenem (Fig. 2), which have a penicillin-like five-membered ring, but the sulfur at C-1 in the ring is replaced with a carbon atom, and a double bond exists between C-2 and C-3 (Jeon et al. 2015).

**Fig. 3:**
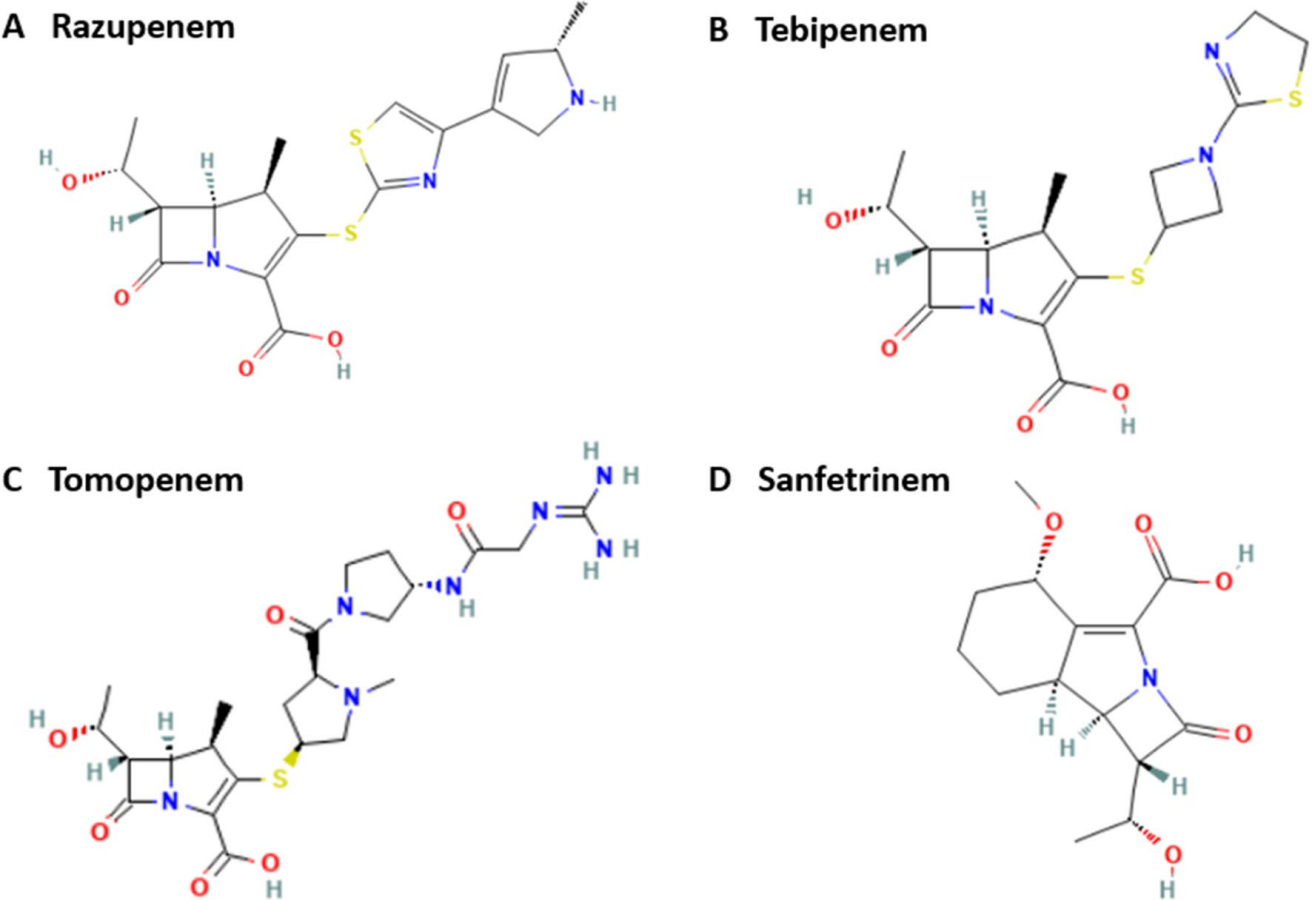
Chemical structure of the newest carbapenem antibiotics: **A** razupenem, **B** tebipenem, **C** tomopenem, and **D** sanfetrinem. Source: PubChem Database with own modifications (https://pubchem.ncbi.nlm.nih.gov/). Created with MS Office

New-generation carbapenems include razupenem, tebipenem, tomopenem, and sanfetrinem (Fig. 3). Tebipenem displays higher selective antibacterial and bactericidal activity, in particular against β-lactamase-non-producing strains. It is highly effective in the treatment of infections caused by ampicillin-resistant *Haemophilus influenzae* and *Neisseria gonorrhoeae*. Tomopenem is also characterized

by broad-spectrum activity, including against methicillin-resistant *Staphylococcus aureus* (MRSA) and *Pseudomonas aeruginosa*. In addition, tomopenem has a longer half-life than other carbapenems, excluding ertapenem. Panipenem is characterized by broad-spectrum activity against both Gram-positive and Gram-negative aerobic and anaerobic bacteria. However, *Pseudomonas aeruginosa* has been found to be resistant to panipenem. This antibiotic is effective in the treatment of respiratory and urinary tract infections. Sanfetrinem is the first antibiotic in a series of novel of tricyclic β-lactam compounds, the trinems. This potent antimicrobial has a broad spectrum of activity. Razupenem is a novel carbapenem antibiotic that has a potential role in the treatment of polymicrobial anaerobic infections (Breilh et al. 2013; El-Gamal et al. 2017).

El-Gamal et al. (2017) proposed the following classification scheme for carbapenems: group 1, broad-spectrum carbapenems which are highly effective in the treatment of community-acquired infections (such as ertapenem), but have limited activity against non-fermenting Gram-negative bacteria; group 2, broad-spectrum carbapenems that effectively target non-fermenting Gram-negative bacteria and are less sensitive to base hydrolysis in solution (imipenem, meropenem, and doripenem); and group 3, carbapenems with clinical activity against methicillin-resistant *Staphylococcus* sp. In recent years, the widespread use of carbapenems has contributed to increased bacterial resistance to these therapeutics. The emergence and spread of acquired resistance to carbapenems is a major public health concern and an indicator of global antibiotic resistance (Aslan and Akova 2022; World Health Organization (WHO), 2018).

### Pathogenicity of *Acinetobacter baumannii* and *Pseudomonas aeruginosa*

New infections that endanger the patients’ health and life pose a significant challenge in human and veterinary medicine. In a report developed by the Centers for Disease Control and Prevention (CDC 2019a), a large percentage of difficult-to-treat infections has been associated with *Pseudomonas aeruginosa* and *Acinetobacter baumannii*. According to a WHO report (2017b), carbapenem-resistant *P. aeruginosa* and *A. baumannii* are critical pathogens that are difficult to eliminate and will be responsible for many deaths in the coming years. *Acinetobacter baumannii*, *P. aeruginosa*, *Escherichia coli*, *Staphylococcus aureus*, *Klebsiella pneumoniae*, and *Streptococcus pneumoniae* belong to a group of priority pathogens that pose the greatest threat to human health and life at present and in the future. In 2019, these pathogens were responsible for 929,000 deaths attributable to antimicrobial resistance and 3–57 million deaths not directly associated with antimicrobial resistance (Murray et al. 2022). *Acinetobacter baumannii* and *P. aeruginosa* alone caused more than 250,000 deaths linked with antimicrobial resistance.

*Acinetobacter baumannii* is an opportunistic pathogen that is resistant to common disinfectants and produces capsular polysaccharides and biofilm that contribute to its pathogenicity (Lima et al. 2020). This pathogen specifically targets moist tissues such as mucous membranes and areas of the skin that become exposed through accident or injury (Howard et al. 2012). In 2017, carbapenem-resistant (CR) bacteria of the genus *Acinetobacter* were responsible for around 8500 infections in the USA, and the associated treatment costs exceeded USD 280 million (CDC 2019b). These pathogens caused mainly lung, wound, blood, and urinary tract infections in ICU patients. Infections caused by CR and/or MDR *A. baumannii* have a high mortality rate (WHO, 2017b). In 2017 alone, CR *Acinetobacter* sp. infections were responsible for more than 700 deaths in the USA. Infections caused by carbapenem-resistant *Acinetobacter baumannii* (CRAB) give particular cause for concern due to the plasticity of bacterial genomes, rapid spread in the hospital environment, and ability to colonize hospital surfaces, including ventilators. These bacteria are responsible for around 47% cases of ventilator-associated pneumonia (VAP) in ICUs (Rezai et al. 2017). In addition, CRAB pathogens are naturally resistant to several classes of antibiotics and easily acquire resistance to new therapeutics (CDC 2019b; World Health Organization (WHO), 2017b).

Carbapenem-resistant *A. baumannii* also pose health risks in veterinary medicine. In a study conducted in 2016–2020, CRAB and CR *Acinetobacter pittii* with various MDR patterns were identified in isolates from canine and feline hosts, which points to clonal proliferation or diversity of these microorganisms. The prevalence of CRAB infections in companion animals has been less extensively studied than the prevalence of infections caused by CRAB and other MDR pathogens in humans. Therefore, the mechanisms underpinning the clonal proliferation of CRAB in companion animals remain fairly unknown (Leelapsawas et al. 2022). In general, many *Acinetobacter* spp. are isolated from domesticated animals and livestock, where these bacteria may be a part of the natural microbiota. However, isolates from animal hosts are characterized by growing levels of antimicrobial resistance, including MDR and CR. According to recent research, *A. baumannii* has evolved into a veterinary nosocomial pathogen (van der Kolk et al. 2019).

*Pseudomonas aeruginosa* is an opportunistic pathogen infects the lungs, blood, urinary tract, endocardium, digestive tract, and surgical sites. These infections are particularly dangerous for patients with chronic lung diseases (CDC 2019b). *Pseudomonas aeruginosa* has several virulence mechanisms that increase its ability to cause severe infections, including toxin secretion, quorum sensing, and biofilm production (Reynolds and Kollef 2021). In 2017, MDR *P. aeruginosa* caused 32,600 infections and 2700 deaths in the USA, and the overall treatment cost exceeded USD 767 million (CDC 2019b). Similarly to CR *A. baumannii*, CR *Pseudomonas aeruginosa* (CRPA) have been recognized as critical pathogens by the World Health Organization (WHO) (2017b), and the growing levels of carbapenem resistance in this group of pathogens compromise the efficacy of the existing treatments and increase the risk of death. A large international study revealed that *P. aeruginosa* was responsible for 16.2% of all infections in ICU patients and caused 23% of all ICU-acquired infections, where the respiratory source, including VAP, was the most common site of *P. aeruginosa* infection (Reynolds and Kollef 2021). According to Ssekatawa et al. (2018), the prevalence of CRPA and CRAB infections in African countries is nearly as high as the prevalence of infections caused by CR Enterobacteriaceae, which illustrates the scale of the problem, particularly in developing countries.

In companion animals, *P. aeruginosa* has been found to cause infections of the external ear canal, superficial skin infections, purulent lesions on the skin and in perianal zones, wound and urinary tract infections, and pyoderma, particularly in dogs (Fernandes et al. 2018; Dégi et al. 2021). Microorganisms responsible for canine skin diseases have to be isolated and identified to make the correct diagnosis and introduce the appropriate treatment. However, *P. aeruginosa* infections are difficult to treat because this pathogen is highly resistant to many first-line antibiotics (Dégi et al. 2021). Despite the fact that carbapenems have not been licensed for use in veterinary medicine, CR *P. aeruginosa* have been isolated from cats and dogs in Romania (Gentilini et al. 2018) and from captive blackbucks and leopards kept in an Indian zoo in 2015–2016 (Vinodh Kumar et al. 2021). In 2016, CR *P. aeruginosa* were isolated from dogs undergoing veterinary treatment as well as from their owners. In addition, CRPA were identified in the owners’ households and in other pets not receiving pharmacological treatment (Fernandes et al. 2018). Similarly to CRAB, CRPA could have also evolved into a veterinary nosocomial pathogen (van der Kolk et al. 2019; Leelapsawas et al. 2022).

According to the literature, domesticated animals and livestock play the key role in the spread of pathogenic bacteria and antibiotic resistance. Antibiotics are often overused in farms, where entire herds or flocks, including uninfected animals, receive antimicrobial treatment to limit the spread of pathogens (Palma et al. 2020). *Acinetobacter baumannii* causes infections in cattle and poses a significant health risk not only for animals, but also for humans (Nocera et al. 2021). *Pseudomonas aeruginosa* is ubiquitous in poultry farms and wild birds. This pathogen is particularly dangerous for newly hatched chicks, where *P. aeruginosa* causes respiratory infections, diarrhea, dehydration, and septicemia and increases mortality. *Pseudomonas aeruginosa* poses a significant health threat for birds, farm personnel, and poultry consumers (Abd El-Ghany 2021). On the global scale, antibiotics are less widely used in livestock farms for therapeutic purposes than for growth promotion and disease prevention (Palma et al. 2020).

### Occurrence of *Acinetobacter baumannii* and *Pseudomonas aeruginosa* in the natural environment

Bacteria of the genera *Acinetobacter* and *Pseudomonas* are cosmopolitan Gram-negative aerobic microorganisms of the class Gammaproteobacteria. They colonize water and soil and can be a part of the normal microbiota in some animals. These opportunistic bacteria cause infections only in immunocompromised hosts (Lupo et al. 2018).

According to the current definition, the genus *Acinetobacter* consists of Gram-negative, strictly aerobic, non-fermenting, non-motile, catalase-positive, oxidase-negative bacteria with a DNA G + C content of 39–47% (Howard et al. 2012). This bacterial genus has undergone many taxonomic changes over the years, and *A. baumannii* was formally designated only in 1986 (Bouvet and Grimont 1986). Despite the fact that earlier accounts of *Acinetobacter* infections in the literature are difficult to interpret, the infections caused by *A. baumannii* became a significant problem in the 1970s, and the importance of *A. baumannii* as a pathogenic organisms continues to increase, mainly, but not only, in the hospital environment (Antunes et al. 2014). These bacteria are less ubiquitous in the natural environment, but they can colonize soil and water due to their ability to produce biofilm (Chin et al. 2018; Whiteway et al. 2022). According to the literature, *A. baumannii* can survive under diverse environmental conditions and persist on various surfaces for long periods of time. These bacteria can proliferate and survive for up to 50 days in aquatic environments, including saline water bodies (Kovacic et al. 2017). According to Dekić et al. (2018), *Acinetobacter* spp. widely colonize seawater, where it may be an opportunistic fish pathogens.

Bacteria of the genus *Pseudomonas* are ubiquitous in the natural environment. *Pseudomonas aeruginosa* is a highly adaptable and metabolically versatile microorganism that has been identified in various environments, including the human body, soil, water, oil-polluted environments, hospitals, on the surface of bathroom and kitchen faucets, and in bathroom and kitchen wastewater, where it produces biofilm (Crone et al. 2020). Rybtke et al. (2015) identified *P. aeruginosa* biofilm in human and rodent lungs, in open wounds, and on the surface of plastic and silicon objects, both *in vivo* and *in vitro.* According to Abd El-Ghany (2021), *P. aeruginosa* are opportunistic bacteria of many avian species under normal environmental conditions, but they can become pathogenic when exposed to stressors. This bacterial species proliferates most rapidly in wet and moist environments (Mielko et al. 2019). *Pseudomonas aeruginosa* has been identified in drinking water, swimming pools, water supply systems, saunas, and natural water bodies filled with both fresh water and seawater. In recreational waters, popular disinfectants were not fully effective in eliminating these microorganisms (Vukić Lušić et al. 2021).

### Origin and prevalence of carbapenem-resistant *Acinetobacter baumannii *and *Pseudomonas aeruginosa*

*Acinetobacter baumannii* and *Pseudomonas aeruginosa* are cosmopolitan microorganisms that have adapted metabolically to survive in various environments. According to the WHO, carbapenems should be reserved as last-resort antibiotics and should be used only to treat infections that are resistant to first-line drugs. Therefore, hospital and clinical environments are regarded as the main reservoirs of CRPA and CRAB (Dekić et al. 2018). Due to the selective pressure exerted by antibiotics used on patients, environmental bacteria can acquire new traits, such as resistance to antimicrobials and disinfectants. These traits are encoded by ARGs and can be transferred to other bacteria via HGT and other mechanisms involving MGEs.

**Fig. 4:**
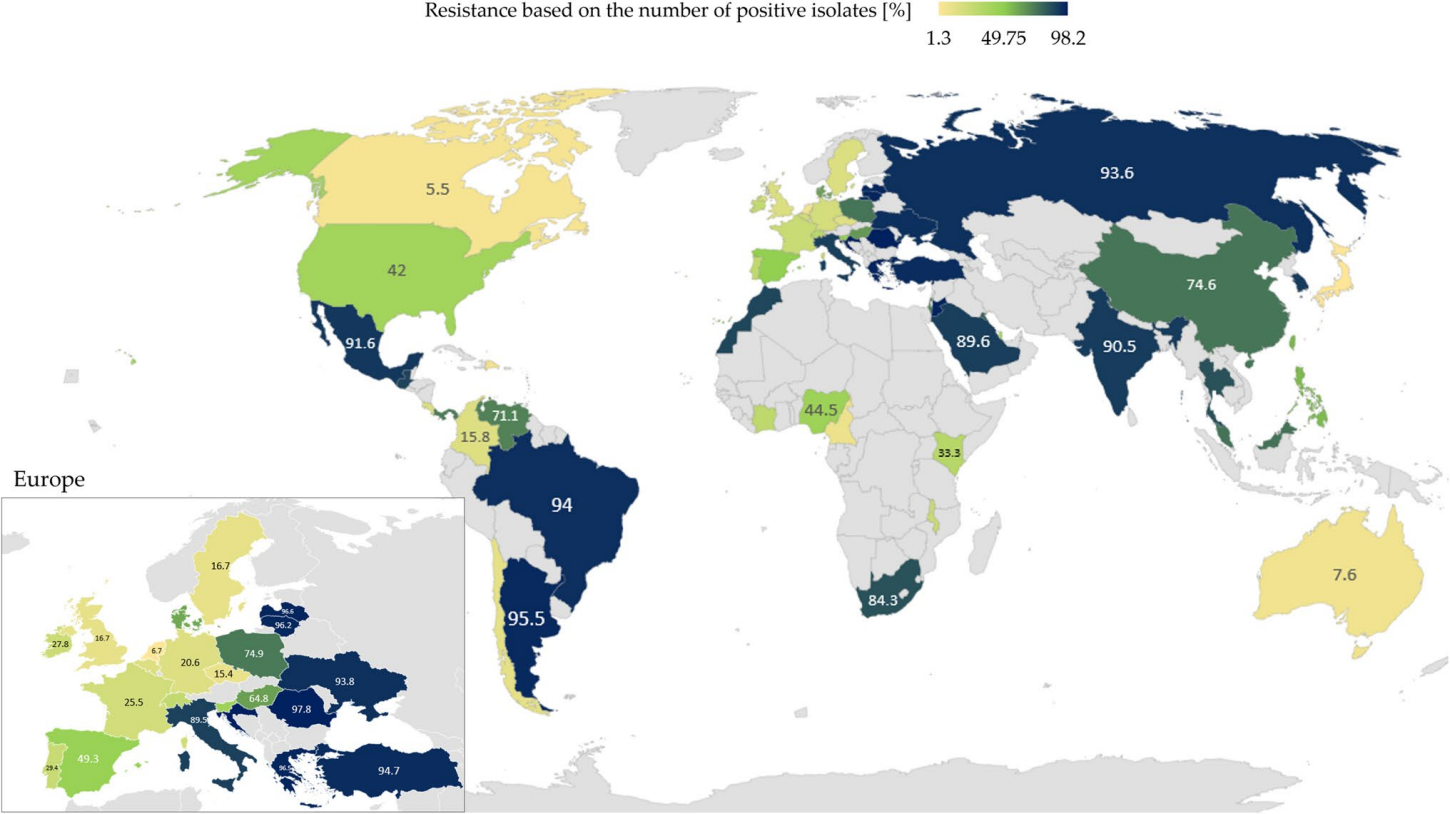
Heatmap of carbapenem-resistant *Acinetobacter baumannii* worldwide in 2019–2022 based on the number of positive isolates among all clinical isolates. Countries with the lowest percentage of resistant strains are marked in yellow; countries with the highest percentage of resistant strains are marked in the darkest navy blue. Gray color denotes the absence of data/cases. Source: Pfizer Surveillance Atlas (https://atlas-surveillance.com/#/heatmap/resistance). Created with MS Office

The number of research articles in PubMed which reported on CRAB increased from a single study in 2000 to more than 266 articles in 2018, which points to the global dissemination of these pathogens. The observed increase can be largely attributed to the transfer of resistant strains both within and between hospitals in the last two decades (Hamidian and Nigro 2019). CRAB have been identified on the surface of medical equipment and facilities and in samples isolated from ICUs, including air, furniture, equipment, and gloves (Raro et al. 2017; Seruga Music et al. 2017). The ability of CRAB to survive on medical equipment and hospital infrastructure determines the rate of which these pathogens spread between hospital staff, patients, and the non-hospital environment. Outbreaks caused by CRAB (Fig. 4) have been reported by civilian hospitals in the USA, Canada, South America, Europe, Africa, Middle East, Southeast Asia, and Australia (Hamidian and Nigro 2019). CRAB pathogens are the main cause of infections in large reference hospitals in South and Southeast Asia, where resistance to carbapenems was determined at 50%, mainly in ICUs (Hsu et al. 2017; Chen et al. 2023). Hospital wastewater also provides a supportive environment for these adaptive bacteria, and it offers a potential route of escape to municipal wastewater and the environment. The above poses a critical health risk for both humans and livestock (Wilharm et al. 2017; Ibrahim et al. 2021).

**Fig. 5:**
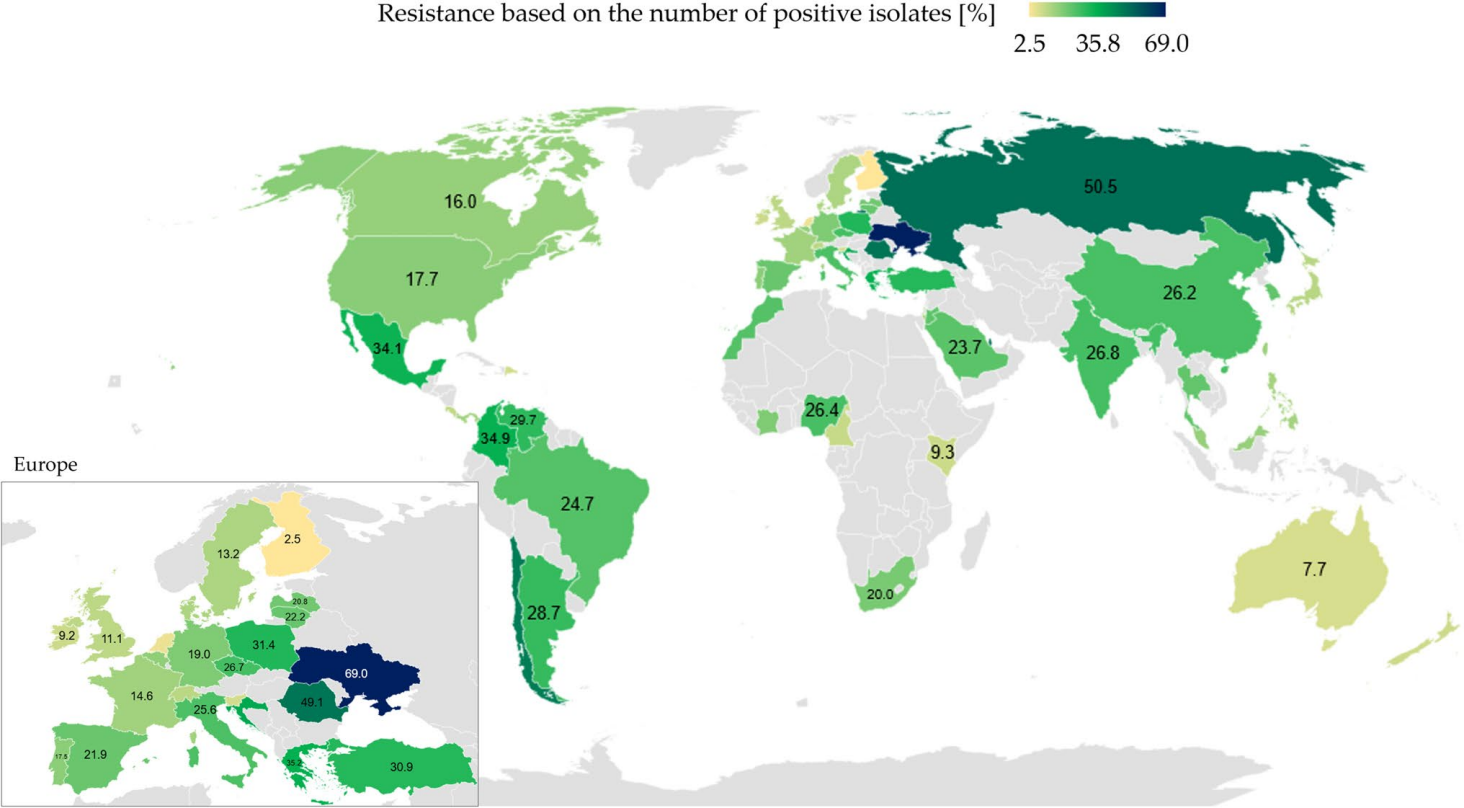
Heatmap of carbapenem-resistant *Pseudomonas aeruginosa* worldwide in 2019–2022 based on the number of positive isolates among all clinical isolates. Countries with the lowest percentage of resistant strains are marked in yellow; countries with the highest percentage of resistant strains are marked in the darkest navy blue. Gray color denotes the absence of data/cases. Source: Pfizer Surveillance Atlas (https://atlas-surveillance.com/#/heatmap/resistance). Created with MS Office

Similarly to *A. baumannii*, CR *P. aeruginosa* are identified in the hospital environment which can act as a reservoir of these pathogens. The prevalence of nosocomial infections caused by CRPA increased globally in recent years. Carbapenem-resistant *P. aeruginosa* are endemic in some countries, including Greece. Carbapenem-resistant and multidrug-resistant *P. aeruginosa* (MDRPA) were first identified in Greece in the 1980s, directly after imipenem and meropenem had been introduced as therapeutics for the treatment of *P. aeruginosa* infections (Karampatakis et al. 2018). Carbapenem-resistant *P. aeruginosa* also pose a problem in many Asian countries (Fig. 5). In Taiwan, the overall prevalence of CRPA in the ICUs of regional hospitals and medical centers was determined at 14 to 20% in 2009–2014. Poor hygiene and sanitation in hospitals and among medical personnel, as well as carbapenem overuse, also contribute to the spread of carbapenem resistance in bacteria (World Health Organization (WHO), 2019).

### Impact of HWW and municipal wastewater on the environment

**Fig. 6:**
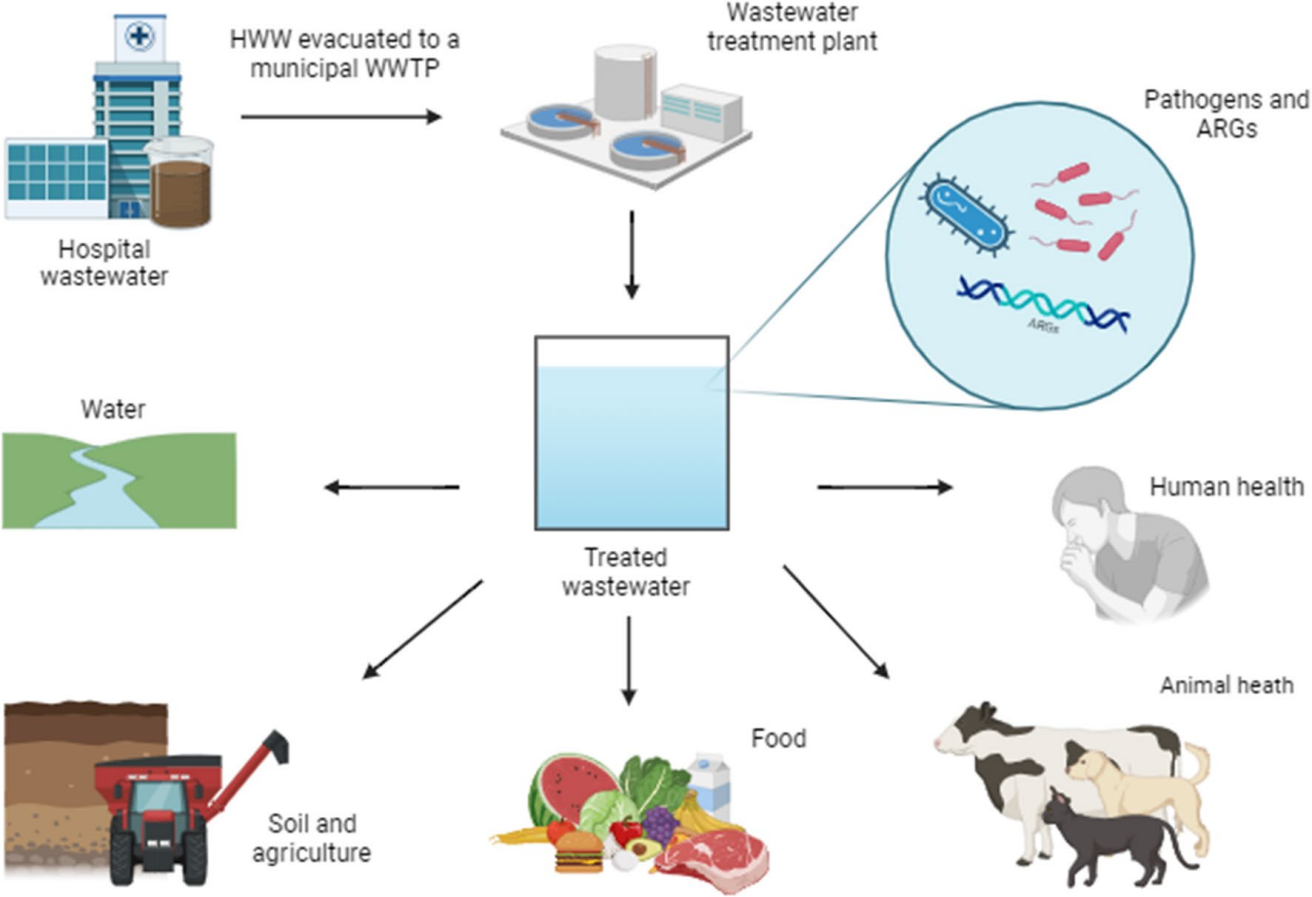
Impact of HWW and municipal wastewater on the environment. Created with BioRender (https://www.biorender.com)

Hospital wastewater (HWW) is evacuated to the municipal sewer system and is treated in wastewater treatment plants (WWTPs), where water is recovered during physical, chemical, and/or biological treatment processes. Wastewater treatment plants remove and/or reduce the concentration of pollutants that are released to the environment with treated wastewater. The last treatment stage may involve UV light and/or chlorine disinfection. However, despite technological progress, dangerous pathogens that pose a threat for humans and the environment are never fully removed in WWTPs (Salgot and Folch 2018). Treated wastewater is evacuated to water bodies in the vicinity, and it can penetrate soil or farmland (Fig. 6). To save water and recycle nutrients, many farms use untreated or partly treated wastewater to fertilize crops that are processed into food and feed. According to Rodriguez-Mozaz et al. (2020), around 15% of farmers around the world fertilize crops with wastewater. Despite the resulting environmental benefits, the use of wastewater as fertilizer carries certain risks. The concentration and type of pathogens and chemical substances in wastewater differ depending on region, sanitation standards, and the socioeconomic status of a given community. Bacterial, viral, and parasite counts in wastewater can be 10–1000 times higher in developing countries than in high-income countries. Depending on the type of contamination and duration of exposure, pathogens can have mild to life-threatening consequences. Diseases caused by pathogenic microorganisms present in wastewater have significantly contributed to premature mortality, particularly in developing countries (Jaramillo and Restrepo 2017).

The spread of pharmaceuticals from treated wastewater has emerged as a critical environmental problem. According to many studies, drug concentrations in water bodies receiving treated wastewater are two to ten times higher downstream the wastewater discharge point. Continuous exposure to low concentrations of some pharmaceuticals can also have unexpected and unintended consequences for animals inhabiting aquatic ecosystems (Aubertheau et al. 2017). Pollutants associated with WWTP products (treated wastewater and sewage sludge) have been identified and classified as emergency pollutants in numerous studies. These substances are highly biologically active, and their physicochemical properties contribute to their persistence and bioaccumulation in the environment. Treated wastewater contains analgesics, hypertension drugs, and antibiotics which can disrupt the hormone metabolism of living organisms. In the past, these compounds were not regarded as pollutants because little was known about their accumulation in soil, water, plant, and animal tissues (Jaramillo and Restrepo 2017). Research studies conducted in countries/regions on six continents provide ample evidence that pharmaceuticals and ARGs are ubiquitous in the environment (Zhuang et al. 2021). Antibiotics such as carbapenems can pose a threat for the environment by promoting the development of antibiotic resistance in natural microbiota and the spread of harmful ARGs (Kim et al. 2021). Męcik et al. (2023) found that antibiotic-resistant *Pseudomonas* sp. persisted in soil for many weeks after introduction and induced transient changes in the microbiological profile of soil.

## Materials and methods

This review article involved a systematic search of the leading scientific databases, such as PubMed, Scopus, and Google Scholar, with the use of specific keywords (Figure S1, Supplementary Materials) associated with CRAB, CRPA, pathogenicity, carbapenem resistance, outbreaks of hospital infections, and the presence of these pathogens in municipal and HWW and in the natural environment. The main aim of this study was to collate information on antibiotic resistance from research studies published in the last decade (Figure S2, Supplementary Materials). Selected research studies and review articles were analyzed, and the results were presented in this manuscript. Only the most recent research findings were analyzed to present the latest trends and challenges relating to antibiotic resistance, CRAB, CRPA, and the transmission of carbapenem resistance genes (CRGs) between hospitals and the natural environment, with special emphasis on the role of HWW in the spread of CRGs in the environment.

## Mechanism of action and resistance to carbapenems

Carbapenems are broad-spectrum antibiotics. With the exception of ertapenem which displays weak activity against *Pseudomonas* and *Acinetobacter baumannii*, carbapenems effectively target both Gram-positive (excluding methicillin-resistant *Staphylococcus aureus*, *Enterococcus faecium*, and *Enterococcus faecalis* which are insensitive to all carbapenems, except imipenem) and Gram-negative bacteria (excluding *Stenotrophomonas maltophilia*). Carbapenems also exhibit activity against anaerobic bacteria. They inhibit the synthesis of the bacterial cell wall by complexing penicillin-binding proteins (PBPs) such as high-molecular-weight enzymes PBP1a, 1b, 2, and 3 (Breilh et al. 2013). PBPs 1a, 1b, 2, and 3 are the main targets of inhibition of further bacterial growth and development, and PBPs 2 and 3 are specific to Gram-negative bacteria. Inhibition of PBP2 induces the formation of spheroplasts without preliminary filamentation and leads to rapid bacterial death. In turn, inhibition of PBP3 generates filamentous forms (Breilh et al. 2013; Aurilio et al. 2022). The position of the side chain is a characteristic feature of carbapenems. The side chain in carbapenems is located in the trans position instead of the cis position which is commonly found in other β-lactams, which makes these antibiotics insensitive to the effects of β-lactamases (Aurilio et al. 2022).

The wide use of carbapenems has increased bacterial resistance to these drugs as well as other antibiotics. Growing levels of bacterial insensitivity to this group of antibiotics has spurred research into carbapenem resistance. In bacteria, carbapenem resistance is associated with the production of enzymes that degrade these antibiotics. β-Lactamases are bacterial enzymes that provide resistance to β-lactam antibiotics, including penicillins, cephalosporins, and carbapenems. Metallo-β-lactamases (MBLs) are a subgroup of β-lactamases that hydrolyze a wide range of β-lactams, including carbapenems that are considered last-resort drugs for the treatment of infections caused by MDR bacteria (Karampatakis et al. 2018; Halat and Moubareck 2022). Metallo-β-lactamases owe their name to the fact that they require a divalent cation, usually a zinc cation, to inactivate antibiotics (Hsu et al. 2017). These enzymes produce four main carbapenemases: class A β-lactamases (such as *Klebsiella pneumoniae* carbapenemase, KPC), two class B β-lactamases (New Delhi metallo-β-lactamases, NDM, and Verona integrin-encoded metallo-β-lactamases, VIM), and class D β-lactamases (such as oxacillinase-48, OXA-48). Other carbapenemase-encoding genes that play a minor role include the Guiana extended-spectrum β-lactamase (GES) and imipenem-hydrolyzing β-lactamase (IMP). Despite different properties and geographic origins, these four main carbapenemases are widely distributed around the world (Zhuang et al. 2021). Bacteria can also become resistant to carbapenems due to mutations that lead to the loss of expression of porin-encoding genes, overexpression of genes encoding efflux pumps, or mutations that induce changes in the production or affinity of penicillin-binding proteins (Oliveira et al. 2021).

### Resistance in *Acinetobacter baumannii*

Various CR mechanisms have been described in *A. baumannii* at the molecular level, including changes in or loss of outer membrane proteins, such as CarO, and modifications of the AdeABC efflux pump (Marchand et al. 2004; Mussi et al. 2011). Although these modifications play a key role in the evolution of carbapenem resistance in *A. baumannii*, they do not induce clinically significant resistance on their own (Coyne et al. 2011). According to recent reports, *A. baumannii* acquire resistance to carbapenems mainly via HGT of ARGs that encode class D (oxacillinase) and class MBL carbapenem-hydrolyzing enzymes. The heterogeneous family of oxacillinase (OXA) enzymes includes several groups of carbapenem-hydrolyzing oxacillinases (OXA-23, OXA-24, OXA-58, OXA-143, OXA-235) and an intrinsic OXA group that is referred to as OXA-Ab for the sake of simplicity. Of those, OXA-23 is most frequently distributed in CRAB in most countries, whereas OXA-24 and OXA-58 are predominant in selected regions. Although OXA-23, OXA-24, and OXA-58 derived from *A. baumannii* belong to class D β-lactamases capable of hydrolyzing carbapenems, their structures differ significantly from other oxacillinases, such as OXA-48—a clinically important carbapenem-hydrolyzing enzyme derived from *Enterobacteriaceae* (Poirel et al. 2010; Hamidian and Nigro 2019). Most CRAB outbreaks around the world can be attributed to the spread of two major clones of CR *A. baumannii*, commonly referred to as global clone 1 (GC1) and global clone 2 (GC2) (Hamidian and Nigro 2019).

One of the genes responsible for carbapenem resistance is *bla*_OXA51-like_ which occurs naturally in *A. baumannii* and is used as a marker of speciation. Many variants of *bla*_OXA-51_ that harbor one or more amino acid substitutions have been identified in the clinical setting. These substitutions can enhance affinity for carbapenems (Chan et al. 2022). However, this gene does not pose a significant threat because it has to be overexpressed to produce CRAB (Nigro and Hall 2018). The *bla*_OXA-23_ gene was first characterized in an *A. baumannii* strain soon after the introduction of carbapenems as therapeutic agents, whereas the *bla*_OXA-58_ gene was first identified in a resistant *A. baumannii* strain with six additional variants. This gene has been linked with hospital outbreaks around the world (Hamidian and Nigro 2019). The *bla*_OXA-24_ gene, initially identified in a CRAB isolate in Spain, has been associated with global hospital outbreaks, and it occurs in many variants that are often found on small plasmids. Other class D carbapenemases, such as OXA-143 and OXA-235, have been linked with CRAB outbreaks in selected countries, but they are less commonly reported and play a minor role in carbapenem resistance in *A. baumannii* (CDC 2019b; Chen et al. 2018; Rodríguez et al. 2018).

### Resistance in *Pseudomonas aeruginosa*

The prevalence of carbapenem resistance and CRPA continues to increase globally. More than 60% of *P. aeruginosa* infections are treated with β-lactams, and resistance to this class of antibiotics (in particular carbapenems) poses a significant clinical problem (Gajdács et al. 2021). *Pseudomonas aeruginosa* can develop resistance to carbapenems due to a combination of β-lactamases (in particular AmpC), porin mutations, overexpression of the MexA-MexB-OprM efflux pump, and/or changes in PBPs (Buehrle et al. 2017). These pathogens have one of the largest bacterial genomes. *Pseudomonas aeruginosa* harbors a combination of genes that have been acquired by HGT and are localized in integrons and MGEs such as transposons, insertion sequences, genomic islands, and plasmids (Halat and Moubareck 2022). The first reports on carbapenem resistance in *P. aeruginosa* emphasized the dominant role of protein inactivation (Opr) in the CRPA phenotype. In addition, resistance to meropenem, but not imipenem, has been linked with MexA-MexB-OprM overexpression (Buehrle et al. 2017). Carbapenemases also play a key role in this process. CRPA strains identified around the world harbor various classes of carbapenemases, mainly metallo-β-lactamases (including VIM, IMP, AIM, SPM, GIM, SIM, DIM, and NDM), KPC, and OXA enzymes (Tada et al. 2017; Karampatakis et al. 2018; Halat and Moubareck 2022). Despite the growing prevalence of CRPA producing mainly MBL, carbapenem resistance can still be caused by the inactivation of OprD (Karampatakis et al. 2018). In CRPA, the production of MBL is encoded mainly by *bla*_VIM-1_, *bla*_VIM-2_, *bla*_VIM-4_, and *bla*_VIM-17_. A combination of several β-lactam resistance mechanisms, including the production of chromosomal AmpC enzymes, efflux pump overproduction, and loss of OprD and/or carbapenemase, can contribute to greater antibiotic resistance than each of these mechanisms alone (Halat and Moubareck 2022).

## Occurrence of carbapenem-resistant strains in the hospital environment

### Case reports of diseases caused by *Acinetobacter baumannii* and *Pseudomonas aeruginosa*

*Acinetobacter baumannii* is an opportunistic pathogen that is associated mainly with the hospital environment. Epidemiological studies are the main source of information about healthcare-related outbreaks of CRAB, and this pathogen poses the greatest threat for ICU patients. The mortality rate associated with *Acinetobacter* infections is high, ranging from 45 to 70% (Bartal et al. 2022). This pathogen is responsible for nearly 5–10% of VAP cases in ICUs and 61% of VAP-related deaths in ICUs. *Acinetobacter baumannii* is naturally resistant to first-line antibiotics for pneumonia treatment, which poses a significant problem in clinical practice (Rezai et al. 2017). The prevalence of healthcare-associated infections caused by CR *Acinetobacter* spp. continues to increase in Europe. On the global scale, these pathogens pose the greatest clinical threat in the Middle East, Europe, East Asia, and Latin America (Table 1). The growing prevalence of Gram-negative bacteria resistant to carbapenems and other antimicrobials has been recognized as a critical challenge by healthcare professionals and governments in many South and Southeast Asian countries (Lamba et al. 2017; Hsu et al. 2017; Liu et al. 2020). During the COVID-19 pandemic, the prevalence of CRAB outbreaks increased because many patients possessed potential risk factors for VAP, including hypertension, chronic obstructive pulmonary disease, chronic renal failure, prolonged ICU stays, organ failure, and low blood oxygenation (Gottesman et al. 2021; Polly et al. 2022). The number of published research articles on clinical outbreaks of CRAB increased by nearly 85% in the last 5 years, from 1919 in 2006–2011 to 3532 in 2012–2017. These observations point to growing levels of carbapenem resistance in *A. baumanni*i in various medical sectors, including in hospital units treating patients with burn injuries (Lima et al. 2019).

**Table 1:** Prevalence of carbapenem-resistant clinical isolates of *Acinetobacter* spp. and *Acinetobacter baumannii* from hospital patients in the most endangered countries in 2012, 2017, and 2021. Source: Surveillance Atlas of Infectious Diseases of the European Center for Disease Prevention and Control (ECDC) (https://atlas.ecdc.europa.eu) and Pfizer Surveillance Atlas ()

Region	Country	Genus/species	R - resistant isolates [*N*]			References
			2012	2017	2021	
Africa/Middle East	Egypt	*Acinetobacter baumannii*	16	-	-*	Pfizer Surveillance Atlas (https://atlas-surveillance.com)
	Israel	*Acinetobacter baumannii*	45	-	114	
	Jordan	*Acinetobacter baumannii*	15	14	46	
	Kuwait	*Acinetobacter baumannii*	33	14	136	
	Morocco	*Acinetobacter baumannii*	32	14	122	
	Nigeria	*Acinetobacter baumannii*	-	-	16	
	Saudi Arabia	*Acinetobacter baumannii*	13	-	24	
	South Africa	*Acinetobacter baumannii*	47	7	132	
Asia	China	*Acinetobacter baumannii*	62	18	417	Pfizer Surveillance Atlas (https://atlas-surveillance.com)
	India	*Acinetobacter baumannii*	-	-	415	
	Malaysia	*Acinetobacter baumannii*	-	-	109	
	Philippines	*Acinetobacter baumannii*	9	4	32	
	South Korea	*Acinetobacter baumannii*	6	14	238	
	Taiwan	*Acinetobacter baumannii*	24	16	129	
	Thailand	*Acinetobacter baumannii*	18	11	220	
Europe	Bulgaria	*Acinetobacter* spp.	35	74	169	ECDC Surveillance Atlas of Infectious Diseases (https://atlas.ecdc.europa.eu)
	Croatia	*Acinetobacter* spp.	-	200	405	
	Cyprus	*Acinetobacter* spp.	13	38	199	
	Greece	*Acinetobacter* spp.	1101	1038	1335	
	Hungary	*Acinetobacter* spp.	201	188	1199	
	Italy	*Acinetobacter* spp.	192	683	2376	
	Poland	*Acinetobacter* spp.	81	232	683	
	Romania	*Acinetobacter* spp.	44	159	361	
	Slovakia	*Acinetobacter* spp.	-	38	82	
North and South America	Argentina	*Acinetobacter baumannii*	59	19	116	Pfizer Surveillance Atlas (https://atlas-surveillance.com)
	Brazil	*Acinetobacter baumannii*	15	16	228	
	Guatemala	*Acinetobacter baumannii*	-	25	56	
	Mexico	*Acinetobacter baumannii*	56	21	132	
	Panama	*Acinetobacter baumannii*	10	5	44	
	USA	*Acinetobacter baumannii*	256	20	184	

*No data/no cases

Hospital infections caused by *P. aeruginosa* pose a considerable challenge in the healthcare sector. These pathogens can cause severe and life-threatening infections, including pneumonia, bacteremia/sepsis, skin and soft tissue infections associated with burns and surgical procedures, otitis externa, keratitis, and urinary infections, and they pose a particular threat for immunocompromised individuals and ICU patients (Gajdács et al. 2021). Sepsis caused by CRPA is one of the three most life-threatening diseases that are very difficult to treat. The prevalence of CRPA outbreaks was found to be highest in ICUs, with a mortality rate of up to 32.8% (Falcone et al. 2023). In a US study analyzing the prevalence of CRPA infections in 1999–2012, CRPA were most common in the intensive care setting (57.0%), in respiratory specimens (61.4%), and in children aged 1–5 years (37.3%) (Logan et al. 2017). The prevalence of CRPA in ICUs in Taipei was determined in the range of 15 to 23% (Tsao et al. 2018). Infections caused by multidrug-resistant *P. aeruginosa* (MDR-PA), including CR strains, account for two-thirds of all MDRA-PA cases in ICUs and affect 25 out of 1000 hospitalized adults despite strict hand hygiene and infection control. A typical case scenario involved adults who were ventilated via a tracheostomy for 2 weeks. In these cases, the respiratory tract was the main clinical site of infection (Borgatta et al. 2017). Shortly after the outbreak of the COVID-19 pandemic, the prevalence of CRPA infections increased in patients at higher risk of VAP (Loyola-Cruz et al. 2023). Carbapenem resistance in *P. aeruginosa* varies across countries and is highest in Europe, South and Southeast Asia, and Latin America (Table 2). Between 2015 and 2019, the greatest differences in CR were reported in the Asia-Pacific region. Carbapenem resistance rates were lowest in Australia (6.5%) and highest in India (29.3%) (Lee et al. 2019; Kresken et al. 2020).

**Table 2:** Prevalence of clinical isolates of carbapenem-resistant *Pseudomonas aeruginosa* from hospital patients in the most endangered countries in 2012, 2017, and 2021. Source: Surveillance Atlas of Infectious Diseases of the European Center for Disease Prevention and Control (ECDC) (https://atlas.ecdc.europa.eu) and Pfizer Surveillance Atlas ()

Region	Country	Genus/species	R - resistant isolates [N]			References
			2012	2017	2021	
Africa/Middle East	Israel	*Pseudomonas aeruginosa*	17	38	39	Pfizer Surveillance Atlas (https://atlas-surveillance.com)
	Jordan	*Pseudomonas aeruginosa*	4	10	11	
	Kuwait	*Pseudomonas aeruginosa*	18	122	47	
	Morocco	*Pseudomonas aeruginosa*	9	2	62	
	Nigeria	*Pseudomonas aeruginosa*	-*	-	34	
	Qatar	*Pseudomonas aeruginosa*	-	-	42	
	South Africa	*Pseudomonas aeruginosa*	28	101	43	
Asia	China	*Pseudomonas aeruginosa*	53	142	245	Pfizer Surveillance Atlas (https://atlas-surveillance.com)
	India	*Pseudomonas aeruginosa*	-	-	249	
	Japan	*Pseudomonas aeruginosa*	34	20	36	
	Philippines	*Pseudomonas aeruginosa*	29	84	46	
	South Korea	*Pseudomonas aeruginosa*	10	67	130	
	Taiwan	*Pseudomonas aeruginosa*	21	31	61	
	Thailand	*Pseudomonas aeruginosa*	19	91	84	
Europe	Austria	*Pseudomonas aeruginosa*	82	101	117	ECDC Surveillance Atlas of Infectious Diseases (https://atlas.ecdc.europa.eu)
	Croatia	*Pseudomonas aeruginosa*	57	73	67	
	Czechia	*Pseudomonas aeruginosa*	74	61	97	
	France	*Pseudomonas aeruginosa*	310	238	467	
	Germany	*Pseudomonas aeruginosa*	47	238	425	
	Greece	*Pseudomonas aeruginosa*	433	323	192	
	Hungary	*Pseudomonas aeruginosa*	170	268	421	
	Italy	*Pseudomonas aeruginosa*	171	285	773	
	Poland	*Pseudomonas aeruginosa*	39	95	123	
	Portugal	*Pseudomonas aeruginosa*	116	222	143	
North and South America	Argentina	*Pseudomonas aeruginosa*	51	80	77	Pfizer Surveillance Atlas (https://atlas-surveillance.com)
	Brazil	*Pseudomonas aeruginosa*	16	98	112	
	Chile	*Pseudomonas aeruginosa*	30	102	120	
	Colombia	*Pseudomonas aeruginosa*	17	92	190	
	Mexico	*Pseudomonas aeruginosa*	57	161	141	
	USA	*Pseudomonas aeruginosa*	244	23	265	
	Venezuela	*Pseudomonas aeruginosa*	26	81	46	

*No data/no cases

### Hospitals as an environment conducive to the spread of carbapenem-resistant microorganisms

Due to their persistence in the hospital environment, *A. baumannii* are exposed to antibiotic selective pressure, which not only leads to the rapid acquisition of resistance determinants, but also contributes to the emergence of highly resistant *A. baumannii* strains (Raro et al. 2017). In addition to patients, potential sources of CRAB in hospitals include supplies (carts and protective masks), invasive equipment (catheters), measuring equipment (blood pressure cuffs), furniture and fittings (door handles, bed rails, linens, mattresses, pillows, sinks), ventilatory and respiratory equipment, air conditioners, infusion pumps, and liquids (blood products, enteral nutrition formulas, saline, soap, distilled water, humidifier water, or non-sterile water) (Nguyen and Joshi 2021). Medical personnel can also unwittingly spread CRAB to patients and premises. In a study analyzing a CRAB outbreak in a Korean ICU, 10.9% of medical personnel’s hands were colonized with these pathogens (Choi et al. 2010). Longitudinal studies may be helpful in identifying the modes of CRAB transmission and designing appropriate measures to prevent and control the spread of infections caused by these pathogens. (Ng et al. 2018). Many hospital CRAB strains participate in the transfer of CRGs via MGEs, which promotes the spread of carbapenem resistance to other bacteria. As a result, carbapenemases are produced by bacterial cells that previously did not possess such an ability (Cai et al. 2017).

Research has shown that in addition to direct transmission between patients and between patients and medical personnel, the hospital environment plays a key role in the spread of pathogens, including CRPA (Chia et al. 2020). Patients infected with CR *P. aeruginosa* are at greater risk of delayed antimicrobial treatment, which can prolong hospitalization and increase the risk of subsequent infections caused by antibiotic-resistant microorganisms, morbidity, and mortality (Spagnolo et al. 2021). Büchler et al. (2023) analyzed the prevalence of CRPA in healthcare workers (HCW) in a review article. In the total number of 37 studies where HCW were screened, seven studies (18.9%) reported positive CRPA screenings in HCW (1 to 34 HCW per outbreak investigation), 23 studies (62.2%) reported HCW isolates that were not identical to the isolate of the index patient, and eight studies (21.6%) reported identical isolates (1 to 27 identical isolates per outbreak investigation). Aruhomukama et al. (2019) found that the prevalence of CRAB and CRPA in the ICU of the Mulago Hospital was low in 2015-2017, but carbapenemase genes, in particular *bla*_VIM_ and *bla*_OXA-23_, were highly prevalent in these strains. In the hospital environment, the main sources of *P. aeruginosa* that are not associated with patients or HCW include air, drinking water, faucets, sink surfaces, and sewer systems. These pathogens are evacuated with wastewater to WWTPs, and CRGs can be transmitted to the environment via MGEs and HGT (Kizny Gordon et al. 2017; Inda-Díaz et al. 2023).

Hospitals deserve closer attention as the key reservoirs of highly resistant pathogens and potential outbreak sites. The guidelines of the European Society of Clinical Microbiology and Infectious Diseases (ESCMID) recommend cleaning and disinfection of the hospital environment, but do not make any recommendations regarding environmental screening to identify potential sources of infection (Tacconelli et al. 2014; Büchler et al. 2023). Adequate prevention and control policies that involve all HCW should are thus urgently needed (Medioli et al. 2022; World Health Organization (WHO), 2019). Longitudinal screening studies of patients in environments at high risk of CRAB outbreaks, combined with advanced diagnostic methods such as whole-genome sequencing (WGS), are powerful tools for screening nosocomial transmission of CRAB and controlling the spread of these infections (Ng et al. 2018). Tomczyk et al. (2019) reviewed studies proposing various interventions, including contact precautions, active surveillance of isolates, monitoring, auditing and reporting on the introduced precautionary measures, patient isolation or cohorting, hand hygiene, and environmental cleaning. A significant decrease in morbidity and/or prevalence of infections was reported in nearly all studies emphasizing the importance of these intervention components.

## Hospital and municipal wastewater

### Hospital wastewater as a hotspot of ARB and ARGs

Due to their specific parameters and composition, HWW can act as hotspots of ARB, in particular MDR Gram-negative bacteria that harbor many ARGs and are increasingly often identified in HWW (Rozman et al. 2020). High concentrations of pharmaceuticals, hormones, contrast agents, legal and illegal substances, and their metabolites can be found in wastewater from various healthcare facilities (Mackull’ak et al. 2021). Antibiotic residues, including residues evacuated with urine and feces, accumulate in sanitary installations in hospitals, including in shower and washbasin siphons and toilets (Sib et al. 2019).

Legal regulations regarding the classification and treatment of HWW differ across countries. The guidelines for the treatment of HWW in Denmark, Greece, Italy, Iran, Taiwan, Korea, Ethiopia, Saudi Arabia, India, Nepal, and Vietnam have been rarely examined in the literature (Kumari et al. 2020). In India, waste management regulations describe the parameters of wastewater generated by hospitals, including hospitals that are connected to the public sewer system, but are not equipped with on-site treatment facilities, as well as hospitals that dump wastewater in the open (Khan et al. 2020; Kumari et al. 2020). In many cases, local WWTPs do not cater to the existing demand. For example, only 40% of wastewater is treated in New Delhi, and ARB and ARGs that are evacuated to municipal sewers with HWW pose a particular risk due to the absence of adequate urban infrastructure. This risk is particularly evident in New Delhi hospitals where antibiotic consumption is often high (Lamba et al. 2017). The prevalence of intestinal diseases and cancers has increased in China and Japan, and in these countries, hospitals are equipped with WWTPs that rely on the conventional activated sludge process. Hospital wastewater is pre-treated before it is evacuated to the public sewer system to prevent the spread of pathogens to the environment (Timraz et al. 2017; Kumari et al. 2020). In European countries, in specific cases, HWW has to undergo preliminary treatment before it is evacuated to the public sewer system to mitigate the risks associated with the large volume of micropollutants in HWW. Disinfection measures are widely applied in practice, including disinfection of wastewater from infectious disease units, blood donation centers, veterinary clinics and hospitals that treat infectious diseases, laboratories that analyze infectious isolates from animals, and municipal WWTPs in response to the sanitation measures implemented by the authorities during epidemics. In Poland, wastewater is not routinely disinfected in mechanical-biological WWTPs that process municipal wastewater. However, foreign practices, in particular American examples, suggest that regular disinfection is justified in certain seasons of the year. In some European countries, wastewater from municipal WWTPs is partly disinfected. In Germany, treated wastewater is disinfected before it is discharged to recreational waters. In France, treated wastewater is disinfected before it is evacuated to protected areas, whereas in Spain, wastewater must be disinfected before it can be used to irrigate farmland, orchards, sports fields, and gardens (Michałkiewicz et al. 2011; Kumari et al. 2020). According to Directive 2008/98/EC of 19 November 2008 on waste (European Commission 2008) and Commission Decision 2000/532/EC of 3 May 2000 establishing a list of hazardous waste (European Commission 2000), some liquid hospital wastes (pharmaceuticals, drugs, solvent residues, soaps, non-chlorinated organic substances) may not be evacuated to the public sewer system, but must be reclaimed and regenerated (Carraro et al. 2018).

Effective treatment and management of HWW is a priority goal due to the growing prevalence of infections caused by ARB and the rapid spread of antimicrobial resistance in wastewater contaminated with ARGs, including CRGs. Hospital wastewater should be evacuated to municipal sewer systems, provided that WWTPs meet regulatory standards and remove at least 95% of all bacteria. Various approaches to wastewater disinfection and sludge removal should also be considered, including methods that rely on advanced solutions such as membrane bioreactors, anaerobic treatment, and oxidation to effectively treat HWW (Top et al. 2020; World Health Organization (WHO), 2017a).

### CRAB and CRPA in hospital wastewater

Due to their ubiquitous nature, CRAB and CRPA are also prevalent in HWW around the world. According to the ECDC, at least one out of three hospitalized patients and one out of two patients undergoing surgical treatment in the EU receive antibiotics each day. Some antibiotic treatments may be redundant and may contribute to the spread of antimicrobial resistance. Many hospitalized patients are infected with resistant bacteria or are asymptomatic carriers of these pathogens (Mackull’ak et al. 2021). In the last decade, numerous studies have demonstrated that antibiotic concentrations in HWW sometimes exceed the minimum inhibitory concentrations (MIC) for microorganisms, which can increase the risk of HWW colonization by ARB (Sib et al. 2019; Voigt et al. 2019). CRAB and CRPA have been frequently identified in HWW in Europe and Asia (Nasri et al. 2017; Ssekatawa et al. 2018; Falodun et al. 2019; Kehl et al. 2022). Kehl et al. (2022) detected CR bacteria, including CRPA, in HWW in Germany. The ST235 *P. aeruginosa* strain is one of the most prevalent carbapenemase producers. This strain evolved in the early 1990s due to the selective pressure of quinolones. ST235 *P. aeruginosa* was implicated in many hospital outbreaks, and it is considered a high-risk strain. A study analyzing HWW in Czechia revealed the presence of CR *P. aeruginosa* that were also resistant to all tested antibiotics. The examined CRPA strains were most resistant to ciprofloxacin, gentamycin, meropenem, ceftazidime, amikacin, piperacillin/tazobactam, and aztreonam, and some strains were classified as MDR (Roulová et al. 2022). In 2016–2018, XDR *A. baumannii* were identified in different departments of a children’s hospital in Zagreb, including in intensive care, burn, surgery, pediatric, and orthopedic units. All tested isolates were resistant to piperacillin/tazobactam, ceftazidime, cefotaxime, ceftriaxone, cefepime, imipenem, meropenem, gentamycin, and ciprofloxacin (Lukić-Grlić et al. 2020), and they were isolated from patients, which can be directly linked with the presence of these pathogens in HWW. CRAB were also identified in HWW in Brazil, China, and South Africa (Kovacic et al. 2017; Anane et al. 2019). In addition, there is evidence to indicate that antibiotics bind to the matrix of bacterial biofilms and are released during stagnation time between subsequent uses of the toilet or washbasin; therefore, antibiotic residues are nearly always present in the sewer system (Sib et al. 2019; Voigt et al. 2019). Previous studies have revealed a low prevalence of carbapenemase genes in clinical bacterial isolates from hospital waste, but at present, they are detected much more frequently (Endraputra et al. 2021). Similar concentrations of *bla*_NDM_ and *bla*_OXA-48_-like genes were determined in wastewater from different medical centers in Tunisia. The highest absolute concentration of the studied genes in 1 mL of wastewater was determined at 8.30×10^6^ copies for *bla*_NDM_ in the Regional Hospital in Sidi Bouzid and at 6.90×10^6^ copies for *bla*_OXA-48_ in the Regional Hospital in Gafsa. In turn, the concentration of *bla*_KPC_ reached 9.31×10^4^ copies in 1 mL, and this gene was identified in only three out of the seven analyzed wastewater samples (Nasri et al. 2017). In the above study, *A. baumannii* isolated from fecal samples harbored CRGs such as *bla*_VIM_, *bla*_OXA-23_, and *bla*_IMP_, which were evacuated with HWW. These findings suggest that the genetic material (including ARGs) of microorganisms colonizing patients and HWW can be spread via HGT (Nazari et al. 2022).

### Risk of CRAB and CRPA dissemination from municipal wastewater

In the last decade, CRAB and CRPA have been identified in environments that are subjected to pressure from municipal wastewater. Municipal waste represents the largest portion of human-generated waste. In WWTPs, municipal wastewater is processed with the use of various technologies to prevent pathogens from reaching the natural environment. Municipal wastewater combines various types of sewage generated in cities, including household, industrial, hospital, and storm wastewater. Hospital wastewater is regarded as the main source of clinically relevant CR bacteria (Higgins et al. 2018; Hrenovic et al. 2017a, b). In WWTPs that process HWW, CRB have been identified in both influent and effluent wastewater. The average counts of CRB in 1 mL of influent and effluent wastewater were 3.5 log and 1.3 log CFU, respectively, and CRB accounted for 47% and 26% of total heterotrophic bacteria in these samples, respectively. The concentration of ARB varied across samples and sampling seasons (Hrenovic et al. 2017a, b). Studies examining the prevalence and diversity of ARB in municipal wastewater provide information about where and to what extent clinically relevant pathogens are released into the environment. When discharged into the environment, ARB may be prevalent in soil, plants, and surface water; they can colonize humans, companion animals, and livestock, contaminate food and food products, and pose a serious risk for public health (Cirkovic et al. 2023). The vast majority of potentially pathogenic CRB rely on the acquisition and expression of ARGs as the main resistance mechanism, whereas potentially pathogenic environmental CRB develop resistance to many antimicrobials through intrinsic mechanisms that act alone or in combination (Oliveira et al. 2021). The chemical and physical properties of wastewater, its composition (including the proportions of municipal and hospital wastewater), the applied treatment methods, and processing conditions (such as differences in hydraulic retention time, HRT) can increase selective pressure by enabling bacteria to acquire new traits (including resistance to carbapenems) via HGT (Kumari et al. 2020; Hutinel et al. 2021). Exposure to HWW can increase the relative abundance of transconjugates, thus contributing to the emergence and transmission of resistant pathogenic and environmental bacteria (Hutinel et al. 2021). Bacterial populations, including CRB, are significantly reduced, but not fully eliminated during wastewater treatment (Hrenovic et al. 2017a); therefore, more effective methods of wastewater treatment are needed, which poses a considerable technological challenge.

## Occurrence of CRAB and CRPA in the natural environment

Due to the growing prevalence of CRB in the hospital environment, hospital and municipal wastewater, and veterinary treatment waste, these microorganisms also affect the natural environment. Hospital environment and HWW play an important role in contaminating soil and water bodies with CRAB, CRPA, ARGs, and carbapenem antibiotics. Every year, large quantities of these resistant pathogens and genetic elements are introduced into the environment through hospital waste disposal practices (Kumari et al. 2020). Moreover, contribution from veterinary waste also adds to the environmental burden of antimicrobial resistance. Residues from treatments of companion animals and livestock contribute to the contamination of soil and water bodies with these resistant pathogens, genetic elements and antibiotics (Męcik et al. 2023). CRAB and CRPA constitute a large group of microorganisms that have been classified as critical priority pathogens by the WHO (WHO, 2017b). These pathogens and CRGs are evacuated to local water bodies and soil with treated wastewater, and they indirectly reach the food chain. Pathogens, pharmaceuticals, and ARGs present in wastewater influence diverse microbiota and contribute to the spread of carbapenem resistance via MGEs and HGT, which poses a considerable threat for human and animal health. The distribution of CRAB and CRPA in the natural environment and the presence of CRGs in the natural microbiota of the discussed environments are presented in Table 3.

**Table 3:** Occurrence of CRAB, CRPA, and carbapenem resistance in the natural microbiota of aquatic, soil, and food environments

Environment	Research site	Microorganism	Resistance*	Gene**	References
I. Aquatic	Lake	*Enterobacter* sp.	IMI, MER	*bla*_IMI-2_	(Harmon et al. 2019)
	Flood control reservoir	*Pseudomonas alcaligenes*	IMI, MER	-	
		*Stenotrophomonas* sp.	CTA, IMI, MER, TET	*bla*_L1_	
	Natural pool filled with rain run-off	*Stenotrophomonas* sp.	CTA, GEN, IMI, MER, TET	*bla*_L1_	
	Park lake	*Pseudomonas geniculata*	GE, IMI, MER	-	
		*Stenotrophomonas* sp.	CTA, GEN, IMI, MER, TET	*bla*_L1_	
	River near WWTPs	*Acinetobacter baumannii*	3GC, 4GC, CR, MDR, PEN, XDR	*bla*_OXA-48_, *bla*_NDM,_ *bla*_TEM,_ *bla*_SHV,_ *bla*_CTX_	(Sahoo et al. 2023)
	Downstream river	*Acinetobacter baumannii*			
	River	*Acinetobacter baumannii*	CR	-	(Serwecińska et al. 2021)
	Wastewater	*Acinetobacter baumannii*	CR	-	
	River	*Acinetobacter baumannii*	CR, MDR	*bla*_OXA-51_, *bla*_OXA-23_	(Turano et al. 2016)
		*Pseudomonas aeruginosa*	CR, MDR	*bla*_SPM-1 2/8/10_ *bla*_SPM-1 10/1/11_ *bla*_SPM-1 22/3/11_	
	Swimming pool	*Pseudomonas aeruginosa*	IMI, MDR, MER, PIP	*opr*D, *bla*_KPC_, *bla*_NDM_, *bla*_IMP_, *bla*_OXA-48-like_, *bla*_VIM_	(Schiavano et al. 2017)
	Healthcare facilities				
	Receptive facilities				
	Municipal waterworks				
	Forest water	*Stenotrophomonas maltophilia*	AZT, CTA, ERT, IMI	-	(Djenadi et al. 2018)
	Borehole water	*Klebsiella* sp.	AZT, CTZ, CTA, ERT, IMI, MER	-	
		*Pseudomonas* sp.	AZT, CTZ, CTA, ERT, IMI, MER	-	
		*Enterobacter* sp.	AZT, ERT, IMI	-	
		*Serratia* sp.	AZT, CTZ, CTA, ERT, IMI, MER	-	
		*Morganella* sp.	AZT, CTZ, CTA, ERT, IMI, MER	*galE*	
	Coastal marine environment	*Rheinheimera* sp.	3GC, CR, PEN	-	(Dewi et al. 2020)
		*Variovorax* sp.	3GC, CR, PEN	*bla*_NDM-5_	
II. Soil	Agricultural soil	*Achromobacter* sp.	CTA, MER	-	(Lopez et al. 2020)
		*Cupriavidus* sp.	CTA, GEN, IMI, MER	-	
		*Enterococcus* sp.	CTA, GEN, IMI, MER	-	
		*Planomicrobium* sp.	CTA, GEN, IMI, MER, TET	-	
		*Pseudomonas* sp.	CTA, GEN, IMI, MER, TET	-	
		*Stenotrophomonas* sp.	CTA, GEN, IMI, MER, TET	-	
	Urban soil	*Bradyrhizobium* sp.	CTA, GEN, IMI, MER, TET	-	
		*Cupriavidus* sp.	CTA, GEN, IMI, MER	-	
		*Enterococcus* sp.	CTA, GEN, IMI, MER	-	
		*Pseudomonas* sp.	CTA, GEN, IMI, MER, TET	-	
		*Stenotrophomonas* sp.	CTA, GE, IMI, MER, TET	-	
	Soil in cattle farms	*Campylobacter* sp.	CR, SUL, TET	*sull, KPC, tetQ, intI1*	(Mills et al. 2021)
		*Salmonella* sp.			
	Rhizosphere soil	*Morganella* sp.	CTZ, CTA, ERT, IMI, MER	*bla*_TEM-116_	(Djenadi et al. 2018)
		*Pseudomonas* sp.	AZT, CTZ, CTA, ERT, IMI, MER	-	
		*Ochrobactrum* sp.	AZT, CTZ, CTA, ERT, MER	-	
		*Stenotrophomonas* sp.	AZT, CTA, ERT IMI, MER	-	
	Salty soil	*Stenotrophomonas maltophilia*	CTZ, CTA, ERT, IMI	-	
		*Serratia marcescens*	CTZ, CTA, ERT, IMI	-	
	Soil in broiler chicken farms	CRB	COL, CR, ESBL	*mcr-1, bla*_NDM_, *bla*_KPC_, *bla*_OXA-1like_, *bla*_CTX-M-9like_, *bla*_TEM_, *bla*_CTX-M-15like_	(Shi et al. 2021)
	Soil in layer chicken farms	CRB	COL, CR, ESBL	*mcr-1, bla*_NDM_, *bla*_KPC,_* bla*_OXA-1like_, *bla*_CTX-M-9like_, *bla*_TEM_, *bla*_CTX-M-15like_	
	Soil in pig farms	CRB	COL, CR, ESBL	*mcr-1, bla*_NDM_, *bla*_KPC,_* bla*_OXA-1like_, *bla*_CTX-M-9like_, *bla*_TEM_, *bla*_CTX-M-15like_	
	Agricultural soil	*Citrobacter sedlakii*	AMC, AMP, CTZ, CTA, CIP, IMI, MER, TET	*bla*_NDM-5_*, bla*_CTX-M-14_*, bla*_SED-1_*, bla*_*O*XA-1_*, bla*_OXA-10_*, arr-3, strA, strB,* *aadA2, aph(30)-Ic, aph(4)-Ia, aac(3)-IVa, aadA1, sul1, fosA, mph(A),* *qnrS1, dfrA12, dfrA14, tet(A), catB3, cmlA1, floR, aac(60)Ib-cr*	(Zhao et al. 2021)
	River sediment	*Klebsiella pneumoniae*	AMC, AMP, CTZ, CIP, CTA, IMI, MER, TRS, TET	*bla*_NDM-5_*, bla*_DHA-1_*, bla*_SHV-1_*, bla*_OXA-10_*, arr-2, strA, strB, aph(30)-IIa,* *water* *IncX1* *aadA1, qnrS1, oqxA, oqxB, qnrB4, fosA, mph(A), sul1, sul2, dfrA14,* *ColRNAI,* *tet(A), cmlA1, floR* *IncHI1,*	
	Illegal dump waste	*Acinetobacter baumannii*	CR	-	(Hrenovic et al. 2019)
		*Burkholderia* sp.	CR	-	
		*Cupriavidus* sp.	CR	-	
		*Enterobacter* sp.	CR	-	
		*Pseudomonas putida*	CR	-	
	Agricultural soil fertilized with pig manure	*Burkholderia* sp.	CR	-	
		*Enterobacter* sp.	CR	-	
		*Escherichia coli*	CR	-	
III. Food	Leafy vegetables	*Acinetobacter baumannii*	AMC, AMP, CPM, DOR, ERT, GEN, IMI, MER	*bla*_OXA-21,_ *bla*_OXA-51_	(Yigrem et al. 2021)
	Chicken meat	*Pseudomonas aeruginosa*	DOR, IMI, MDR, MER, SUL, TET,	*bla*_OXA-50,_ *bla*_OXA-488,_ *bla*_*OXA-494*_*, *_*blaOXA-395*_*, bla*_*OXA-396*_*, bla*_*OXA-486,*_* bla*_*OXA-4*_*, bla*_*OXA-17*_	(Wu et al. 2023)
	Pork meat				
	Italian bulk tank milk	*Pseudomonas* sp.	AG, FQ, IMI, MER, NF, QN	*-*	(Decimo et al. 2016)
	Fresh fruits and vegetables	*Enterobacteriaceae*	AMC, AMP, CTZ, CTA, CTR, IMI, LEV, MER	*bla*_TEM,_ *bla*_SHV_, *bla*_CMY-2,_ *bla*_KPC_, *bla*_OXA-48,_ *bla*_IMP_, *bla*_VIM_	(Jiménez-Belenguer et al. 2023)
		*Acinetobacter* sp.	AMC, AMP, CTZ, CTA, IMI, LEV, MER		
		*Pseudomonas* sp.	AMC, AMP, CTZ, CTA, CTR, IMI, LEV, MER		
	Meat and meat products	*Enterobacter cloacae*	AMC, AMP, CTA, IMI, MER	*bla*_VIM-1,_ *aacA7, dfrA1, smr*	(Sadek et al. 2021)
		*Pseudomonas aeruginosa*	AMC, AMP, AZT, CTA, CIP, CHL, GE, MER, NA, TET	*bla*_VIM-2,_ *aadB, bla*_OXA-10_, *aacA7, dfrA1, smr*	
	Milk	*Pseudomonas aeruginosa*	GEN, IMI, LEV, MDR, MER, PIT,	*bla*_NDM,_ *bla*_PER_	(Radovanovic et al. 2020)
	Meat carcasses				
	Retail seafood	*Enterobacter cloacae*	CR, MDR	*bla*_IMI−1_	(Janecko et al. 2016)
	Shrimp			*bla*_NDM−1_	

*The most alarming antibiotic resistance in the literature

*3GC*, 3rd generation cephalosporin; *4GC*, 4th generation cephalosporin; *AG*, aminoglycosides; *AMC*, amoxicillin/clavulanate; *AMP*, ampicillin; *AZT*, aztreonam; *CTZ*, ceftazidime; *CEP*, ceporex; *CTA*, cefotaxime; *CIP*, ciprofloxacin; *CHL*, chloramphenicol; *COL*, colistin; *CPM*, cefepime; *CR*, carbapenem-resistant; *CRB*, carbapenem-resistant bacteria; *CTR*, ceftriaxone; *DOR*, doripenem; *ERT*, ertapenem; *FQ*, fluoroquinolones; *GEN*, gentamicin; *IMI*, imipenem; *LEV*, levofloxacin; *MDR*, multidrug-resistant; *MER*, meropenem; *NA*, nacylic acid; *NF*, nitrofurans; *NOR*, norfloxacin; *PEN*, penicillins; *PIP*, piperacillin; *PIT*, piperacillin/tazobactam; *QN*, quinolones; *SUL*, sulfonamides; *TRS*, trimethoprim/sulfamethoxazole; *TET*, tetracycline; *XDR*, extremely drug-resistant

**The most widespread resistance genes in the literature

### Aquatic environment

According to Mancuso et al. (2023), CRB pose a problem not only in the healthcare sector, but also in the natural environment. Aquatic ecosystems are the main hotspots of antimicrobial resistance because inadequately treated municipal and hospital wastewater is evacuated to open bodies of water. MDR bacteria such as *Acinetobacter* and *Pseudomonas* are highly likely to survive in these environments. When exposed to antibiotic residues, these bacteria can develop considerable resistance, in particular to carbapenems and can even become completely resistant to other antibiotics, especially colistin and fluoroquinolones (Adegoke et al. 2020). The above undermines the reliability of the existing indicators for monitoring the sanitary safety of drinking and bathing water. Serwecińska et al. (2021) found that water from presumably safe wells can harbor dangerous pathogens such as *Yersinia* which can persist in water for more than seven months. In addition, dangerous pathogens such as *Mycobacterium tuberculosis* and *Helicobacter pylori* can spread with water, which implies that water pollution indicators are insufficient for monitoring the existing threats. Numerous researchers have argued that new, effective intervention strategies should be developed to decrease the prevalence of antibiotic resistance and inhibit the global spread of MDR bacteria (Serwecińska et al. 2021). According to the literature, CRAB and CRPA are increasingly often detected in natural water bodies, including lakes and rivers that receive wastewater effluents (Table 3, I). In Croatia, CRAB strains similar to clinical isolates from a nearby hospital were identified in river water by Seruga Music et al. (2017). Fourteen of the 19 analyzed strains were isolated simultaneously from patients, HWW, municipal wastewater, and river water. These isolates were classified as a single strain, which could suggest that this strain reached the natural environment via HWW. All studied isolates were resistant to carbapenems, fluoroquinolones, aminoglycosides, and penicillins/β-lactams, and they were classified as XDR pathogens. Hubeny et al. (2022) identified CR *Acinetobacter* sp. and *A. baumannii*, as well as CRGs in samples of municipal wastewater and river water. Harmon et al. (2019) reported on the presence of CR *Pseudomonas* in ponds and lakes in Los Angeles, USA, and found that these pathogens were the second largest group of CRB in the region. Interestingly, CR *Pseudomonas* isolates, including *P. geniculata* and *P. otitidis*, were highly abundant in Portuguese rivers, but they were not identified in the Midwestern United States (Harmon et al. 2019). In Italy, *P. aeruginosa* (including CRPA) were detected in drinking water and swimming pools, which indicates that these environments can act as reservoirs of these pathogens (Schiavano et al. 2017). Djenadi et al. (2018) identified CRB in natural water bodies in forests, in flooded boreholes, and in the municipal sewer system in Béjaïa, Algeria.

### Soil ecosystems

Soil is the most biodiverse habitat on Earth and one of the key components of the natural environment. Soil-dwelling microorganisms deliver crucial ecosystem functions and affect human and animal health. The biodiversity of the soil microbiome drives nutrient cycling, decomposition, and plant production, and it decreases pathogen counts in soil (Delgado-Baquerizo et al. 2020). Acquired resistance can modify the soil microbiome and disrupt the ecosystem balance. Numerous research studies have demonstrated that CRGs and CR microorganisms colonize soils around the world (Table 3, II) (Gudeta et al. 2016; Mills et al. 2021). A study conducted in 2020 revealed that soils in Southern California (Los Angeles region) are an underappreciated reservoir of MDR bacteria, including carbapenemase-producing CRB. These microorganisms were more prevalent in agricultural locations than in urban soils, which suggests that farms could play a key role in the transmission of bacteria resistant to carbapenems and other antibiotics (Lopez et al. 2020). Soil-dwelling CRAB are also able to produce biofilm, which can significantly contribute to the spread of carbapenem resistance and the acquisition of CRGs via HGT (Hassan and Khider 2020; Yusuf et al. 2023). In India, CRAB were also identified in samples of urban soil collected far from local hospitals in the Mangaluru region, and these findings can have significant implications for public health (Suresh et al. 2022). A study conducted in Bangladesh in 2015 revealed high counts of MDR *P. aeruginosa* in industrial soil. All MDR isolates were resistant to piperacillin, gentamycin, amikacin, tobramycin, netilmicin, imipenem, doripenem, meropenem, and cefixime. In addition, 60% of the tested isolates had double plasmids with a size of 1000–2000 bp, which suggests that the location of ARGs in MDR *P. aeruginosa* could be correlated with the presence of these plasmids. The above is directly associated with the risk of ARG transmission (Talukder et al. 2021)*.* Hrenovic et al. (2019) identified CRB in soil sampled from illegal dumps of hospital waste, which gives significant cause for concern. In the cited study, clinically relevant bacteria were detected only in soil samples collected from illegal dumping sites, whereas human-associated CRB were not found in soils free from anthropogenic pressure.

### Food contamination

Carbapenem-resistant bacteria pose a considerable challenge in first-line antibiotic therapy. The presence of CRB in food products (Table 3, III) can lead to food poisoning and dangerous infections, particularly in immunocompromised individuals. Consumers have a growing demand for organic, unprocessed foods which are considered to be healthier and of higher quality than conventional products (Batista De Amorim and Nascimento 2017). Despite the above, there is a certain gap in knowledge concerning the microbiological safety of organic foods. Organic foods, in particular organic vegetables, may act as potential reservoirs of antibiotic resistance, and CRB have been identified in many organic farming systems in the last decade (Dahiru and Enabulele 2015; Carvalheira et al. 2021). Jiménez-Belenguer et al. (2023) detected ARB in all samples of organic lettuce, cabbage, strawberries, and spinach. More than 97% of the identified isolates were resistant to at least one antibiotic, and 22.4% of those were insensitive to multiple drugs. In the group of the identified isolates, 14.9% were resistant to all tested β-lactams, and they accounted for 18% of all organic food isolates that harbored *bla*_TEM_ and *bla*_OXA-48_ genes encoding resistance to carbapenems. Meat- and animal-based products are a large part of the human diet worldwide (Sadek et al. 2021). Carbapenem-resistant bacteria were identified in seafood, including shrimp and clams, which indicates that CRGs are prevalent in these products (Janecko et al. 2016; Ramírez-Castillo et al. 2023). Animal-based foods harboring CRAB and CRPA pose a significant threat for the consumers’ health and/or life. CRAB and CRGs were identified in meat and the abattoir environment in New Zealand. The presence of these strains in meat could be attributed to the fact that the abattoir is located in an area where HWW and industrial wastewater are evacuated to water bodies or the fact that antibiotics were misused in the slaughtered animals. As a result, the selective pressure exerted by antibiotics could promote the development of antibiotic resistance in bacteria and the spread of carbapenem resistance (Anane et al. 2019). Antibiotic resistance genes are also commonly found in saprophytic bacteria colonizing fresh dairy products. Many foods contain *Pseudomonas* spp. that can transfer ARGs to human microbiota. These microorganisms are generally associated with food spoilage, but their impact on human health has not been sufficiently investigated. Non-pathogenic *Pseudomonas* strains, such as *P. fluorescens* and *P. putida*, have been implicated in human diseases, but their role remains fairly unknown (Quintieri et al. 2019). *Pseudomonas aeruginosa* produce biofilm on the surface of meat and milk, and these foods should be thoroughly washed and/or thermally processed before consumption (Radovanovic et al. 2020). In addition, the presence of ARGs that can be transferred to normal human and/or food microbiota increases the risk of complicated diseases and requires further research.

## Summary and conclusions

The purpose of this review article was to analyze the presence of CR *Pseudomonas aeruginosa* and *Acinetobacter baumannii* in various environments, especially hospitals and hospital and municipal wastewater as the key reservoirs of these bacteria and CRGs. The growing prevalence of these pathogens and increasing levels of carbapenem resistance pose a serious challenge for public health and environmental protection. The prevalence of infections caused by CRAB and CRPA has increased in the last decade, which indicates that control and preventive measures are urgently needed to de-escalate drug resistance, in particular resistance to last-resort antibiotics. The implementation of rigorous hand hygiene protocols in healthcare facilities appears to be the critical requirement for minimizing the transmission of pathogens between patients and medical personnel. At the same time, infectious outbreaks should be monitored and reported, and trends in antibiotic resistance should be observed. Protocols that encourage responsible use of carbapenems and minimize antibiotic overuse can slow down the emergence of resistance to this group of antibiotics. In addition, innovative protocols for treating HWW are also needed to limit the spread of ARG and ARGs in the natural environment. Hospital wastewater often contains potentially infectious pathogens, and it can act as a significant hotspot of antibiotic-resistant microorganisms. Advanced wastewater treatment technologies can significantly minimize these risks, in line with the One Health approach that recognizes the interconnection between humans, animals, and the environment. International collaboration and the exchange of information enable scientists to monitor global trends and develop effective strategies for controlling antibiotic resistance. Therefore, appropriate measures should be introduced not only at the local, but also at the global level to minimize microbial resistance to antibiotics on all continents. Further research is needed to monitor changes in antibiotic resistance and the efficacy of the implemented interventions.

### Supplementary Information

Below is the link to the electronic supplementary material.

Supplementary file1 (DOCX 416 KB)

## Data Availability

The detailed data used in the review manuscript are available in the authors for the request of the readers.

## Abbreviations

ARB: Antibiotic-resistant bacteria
ARGs: Antibiotic resistance genes
CR: Carbapenem-resistant
CRAB: Carbapenem-resistant *Acinetobacter baumannii*
CRB: Carbapenem-resistant bacteria
CRGs: Carbapenem resistance genes
CRPA: Carbapenem-resistant *Pseudomonas aeruginosa*
HGT: Horizontal gene transfer
HWW: Hospital wastewater
ICU: Intensive care unit
KPC: *Klebsiella pneumoniae* carbapenemase
MBL: Metallo-β-lactams
MDR: Multidrug resistance
MDRPA: Multidrug-resistant* Pseudomonas aeruginosa*
MGE: Mobile genetic elements
MIC: Minimum inhibitory concentration
NDM: New Delhi metallo-β-lactamase
PBP: Penicillin-binding proteins
TDR: Totally drug-resistant
VAP: Ventilator-associated pneumonia
VIM: Verona integron-encoded metallo-β-lactamase
WWTPs: Wastewater treatment plants
XDR: Extremely drug-resistant
