# Supplementary material for: Hospital and municipal wastewater as a source of carbapenem-resistant Acinetobacter baumannii and Pseudomonas aeruginosa in the environment: a review
Source: Environ Sci Pollut Res Int. 2024 Jul 25;31(36):48813–38. doi: 10.1007/s11356-024-34436-x (PMC11310256; doi:10.1007/s11356-024-34436-x)
Supplement: Supplementary file 1 — Supplementary file1 (DOCX 416 KB) [file 11356_2024_34436_MOESM1_ESM.docx]

Supplementary Materials

**Hospital and municipal wastewater as a source of carbapenem-resistant *Acinetobacter baumannii* and *Pseudomonas aeruginosa* in the environment – a review**

Magdalena Męcik^1^, Kornelia Stefaniak^1^, Monika Harnisz ^1^ and Ewa Korzeniewska ^1,^ *

^1^ Department of Water Protection Engineering and Environmental Microbiology, Faculty of Geoengineering, University of Warmia and Mazury in Olsztyn, Prawocheńskiego 1, 10-720 Olsztyn, Poland

***** Corresponding author: [ewa.korzeniewska@uwm.edu.pl](mailto:ewa.korzeniewska@uwm.edu.pl)

**Figure S.1.:** Network of keywords co-occurrence in articles containing the keyword "carbapenem resistance". The size of the nodes is proportional to the frequency of co-occurrence of a given keyword; node colors represent co-occurrence patterns in research articles published in each year of the analyzed period. The network was generated in VOSviewer (v. 1.6.19; 2023).

**
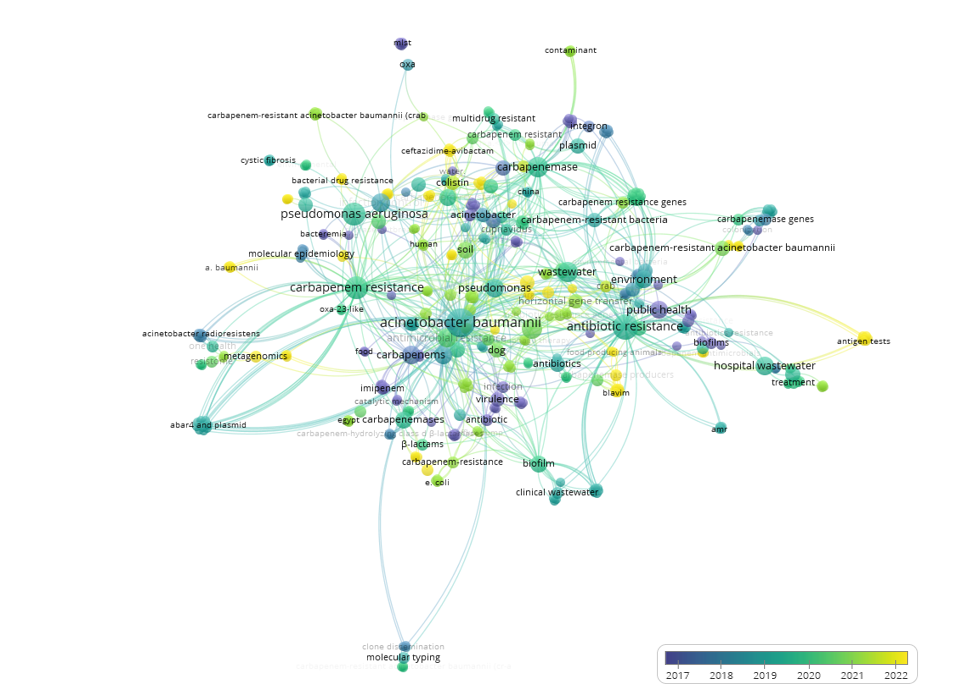
**

**Figure S2**. PRISMA flowchart showing the results of the publication's search and screening process for this review

**Identification of studies via other methods**

**Identification of studies via databases and registers**

Records identified from*:

Databases (n = 300)

Registers (n = 0)

Keywords: antimicrobial resistance, wastewater, hospital wastewater, carbapenem, carbapenemase, carbapenem resistance, Pseudomonas aeruginosa, Acinetobacter baumannii, CRAB, CRPA, soil, food, water

Records identified from:

Websites (n = 2)

Organizations (n = 7)

Citation searching (n = 0)

etc.

Records removed *before screening*:

Duplicate records removed (n = 2)

Records marked as ineligible by automation tools (n = 0)

Records removed for other reasons (n = 15)

**Identification**

Records excluded**

(n = 35)

Records screened

(n = 283)

Reports sought for retrieval.

(n = 248)

Reports assessed for eligibility.

(n =240)

Reports not retrieved

(n = 0)

Reports sought for retrieval.

(n = 9)

Reports not retrieved (n = 8)

**Screening**

Reports excluded:

Reason 1 (n = 0) data

.

Reports assessed for eligibility.

(n = 9)

Reports excluded:

Reason 1 (n = 54) *topic, key words, organism*

Reason 2 (n = 0) *duplicate*

Reason 3 (n = 24) *other*

Studies included in review.

(n = 162)

Reports of included studies

(n = 9)

**Included**

*Consider, if feasible to do so, reporting the number of records identified from each database or register searched (rather than the total number across all databases/registers).

**If automation tools were used, indicate how many records were excluded by a human and how many were excluded by automation tools.

*From:*  Page MJ, McKenzie JE, Bossuyt PM, Boutron I, Hoffmann TC, Mulrow CD, et al. The PRISMA 2020 statement: an updated guideline for reporting systematic reviews. BMJ 2021;372:n71. doi: 10.1136/bmj.n71. For more information, visit: <http://www.prisma-statement.org/>
